# Transcriptional states of CAR-T infusion relate to neurotoxicity – lessons from high-resolution single-cell SOM expression portraying

**DOI:** 10.3389/fimmu.2022.994885

**Published:** 2022-09-28

**Authors:** Henry Loeffler-Wirth, Michael Rade, Arsen Arakelyan, Markus Kreuz, Markus Loeffler, Ulrike Koehl, Kristin Reiche, Hans Binder

**Affiliations:** ^1^ Interdisciplinary Centre for Bioinformatics (IZBI), Interdisciplinary Centre for Bioinformatics, Leipzig University, Leipzig, Germany; ^2^ Bioinformatics Unit, Department of Diagnostics, Fraunhofer Institute for Cell Therapy and Immunology (IZI), Leipzig, Germany; ^3^ Armenian Bioinformatics Institute (ABI), Yerevan, Armenia; ^4^ Research Group of Bioinformatics, Institute of Molecular Biology of the National Academy of Sciences of the Republic of Armenia, Yerevan, Armenia; ^5^ Institute for Medical Informatics, Statistics and Epidemiology, Leipzig University, Leipzig, Germany

**Keywords:** single-cell transcriptomics, CAR-T cell immunotherapy, data portraying, transcriptional states, bioinformatics workflow

## Abstract

Anti-CD19 CAR-T cell immunotherapy is a hopeful treatment option for patients with B cell lymphomas, however it copes with partly severe adverse effects like neurotoxicity. Single-cell resolved molecular data sets in combination with clinical parametrization allow for comprehensive characterization of cellular subpopulations, their transcriptomic states, and their relation to the adverse effects. We here present a re-analysis of single-cell RNA sequencing data of 24 patients comprising more than 130,000 cells with focus on cellular states and their association to immune cell related neurotoxicity. For this, we developed a single-cell data portraying workflow to disentangle the transcriptional state space with single-cell resolution and its analysis in terms of modularly-composed cellular programs. We demonstrated capabilities of single-cell data portraying to disentangle transcriptional states using intuitive visualization, functional mining, molecular cell stratification, and variability analyses. Our analysis revealed that the T cell composition of the patient’s infusion product as well as the spectrum of their transcriptional states of cells derived from patients with low ICANS grade do not markedly differ from those of cells from high ICANS patients, while the relative abundancies, particularly that of cycling cells, of LAG3-mediated exhaustion and of CAR positive cells, vary. Our study provides molecular details of the transcriptomic landscape with possible impact to overcome neurotoxicity.

## Introduction

Chimeric Antigen Receptor (CAR)-T cell therapy is a promising treatment option for patients with B cell lymphomas. It belongs to the family of immunotherapies which share the concept of directing the patients’ immune system against the tumor by releasing breaks of immune regulation and suppression (immune checkpoint inhibition), by boosting an immune response using signaling molecules (cytokine therapy), or by infusion of adapted immune cells to optimally target and dispose the malignant cells (cellular immunotherapy, tumor-infiltrating lymphocytes, and cancer vaccine therapy) (1). Specialized T cells with CAR have proven efficacy in therapy of diverse hematopoietic and lymphatic malignancies such as leukemia (2, 3), myelomas (4, 5) and lymphomas (6–8). CAR-T cells are characterized by receptor proteins that have been engineered to target binding partners as specific as possible for a particular disease, for example CD19 for therapy of B cell lymphoma. Until now, three generations of receptor proteins have been developed, incrementally optimizing the signaling domain of the receptor construct (9, 10).

However, with increasing efficacy of the therapies, immune responses unleashed by immunotherapies cause a series of adverse outcome effects, particularly the cytokine release syndrome (CRS) and neurotoxicity (immune effector cell-associated neurotoxicity syndrome - ICANS) often accompany CAR-T cell therapies (10), rendering risk estimation prior to therapy, safety monitoring and adjunctive treatments indispensable for a save cancer therapy (11). As part of the project imSAVAR (Immune Safety Avatar: nonclinical mimicking of the immune system effects of immunomodulatory therapies; www.imsavar.eu), we aim at identification of potential markers, understanding underlying mechanisms and, in perspective, at providing prediction models for adverse effects of immunomodulatory therapeutics.

Recent studies applied single-cell RNA sequencing to analyses the transcriptome of the infusion products and/or blood samples of patients undergoing anti-CD19 CAR-T cell therapy (12–14). The data set published by Deng et al., 2020 (14) contains 24 infusion product transcriptomes along with information about adverse effects. Their results suggest that heterogeneity in the cellular and molecular features of CAR-T cell infusion products contributes to variation in efficacy and toxicity after therapy of lymphomas. In our re-analysis of this data, we extend the original analyses by an in-detail characterization of the expression landscapes with single-cell resolution and with special focus on T cell subpopulation composition, their transcriptional states, and their relation to neurotoxic side effects. For this, we developed the so-called single-cell data portraying approach by adapting and extending our previous work on self-organizing maps (SOM) portraying of transcriptome, methylome and genome data in bulk settings (15, 16), and its application in the context of cancer (17–20), inflammatory diseases (21–23), and health research (24, 25). The method is based on SOM machine learning (26, 27), and supplements it with extensive analysis and visualization options including pseudo-temporal ordering (28, 29), combined omics studies (30), and proof-of-principle applications in single-cell experiments (31, 32). It is available as R-package ‘oposSOM’ on Bioconductor and GitHub repositories (16), and part of the results of the previous studies are available in an interactive data and results browsing tool (33).

In this publication, we present comprehensive data portraying of the infusion products of CAR-T cell immunotherapy in order to decipher transcriptomic landscapes in terms of defined activation patterns related to the different T cell subpopulations, cellular functions, variations as a function of the CAR construct, and neurotoxicity complications. We will demonstrate capabilities of the single-cell data portraying to disentangle complex co-expression relations of the genes as well as the broad similarity and variability continuum of more than hundred thousand cells. Our analysis revealed that the transcriptional states of cells derived from patients with low ICANS grade do not differ from those of cells from high ICANS patients. On the other hand, their relative abundancies vary markedly thus making relative frequencies of distinct cell states a potential indicator for adverse effects.

## Materials and methods

### Pre-processing of scRNA-Seq data

Single-cell RNA sequencing data of the infusion product of 24 patients undergoing anti-CD19 CAR-T cell therapy was obtained from Gene Expression Omnibus, accession number GSE150992 [(14), see **Table S1** for patient characteristics]. We used the Seurat R-package (34) to process gene counts into expression values. Parameters for quality control and data filtering were adopted from Deng et al. (14). Subsequently, standard Seurat processing workflow was utilized for data integration, normalization and scaling, detection of variable features, t-SNE computation and cell clustering. Please refer to the **Supplementary Material** and (14, 35, 36) for details. Eventually, we obtained data on 20,649 genes in 133,405 cells, which are distributed over 30 clusters.

### T cell subpopulation and calling of cell cycle phases

We applied a set of consensus markers to assign subpopulations of cells derived from the infusion products. CD3 was used as marker gene for T cells, which were subsequently divided into major cell types using CD4 and CD8A/B markers, respectively, and further into naïve, effector, and memory T cells. An overview of all subpopulation markers can be found in the **Supplementary Material**. CAR-positive T cells were defined using the CAR-specific FMC63-CD19scFV marker (14) (see **Supplementary Figure S4A** for fractions of CAR-positive cells in the infusion product samples).

To assign cell cycle status to each cell, we utilized two established gene sets for S phase and for G2M phase, respectively (37), as input for the Seurat cell cycle scoring functionality (34). In result, cell cycle status is obtained for each single cell in terms of G1, G2M, or S phase assignment.

### Down sampling to meta-cells

**Figure 1:**
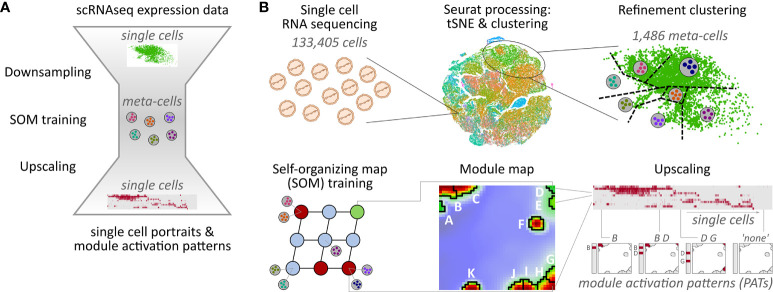
Overview of the downsampling and upscaling processes: **(A)** In short: Single-cell RNA sequencing data is reduced to meta-cell data. After self-organizing map (SOM) training using these meta-cells, data is upscaled to single-cell resolution. **(B)** In detail: scRNAseq data of 133,405 cells was used to generate tSNE and cell clusters using standard Seurat preprocessing workflow. The resulting clusters were then further divided in a refinement clustering step, resulting in 1,486 meta-cells. Expression data of these meta-cells were used for SOM training followed by definition of eleven expression modules A–K Finally, analysis is upscaled to single-cell level by calculating module expression data for each single cell, and stratification of the data by patterns of module activation (PATs).

Self-organizing map (SOM) machine learning was applied for gene clustering, dimension reduction, and multidimensional scaling based on single-cell expression data (16, 31). For this, downsampling was applied to reduce runtime and computer memory requirements of the SOM training. It consists of three steps: The first step is based on unsupervised cell clustering as provided by Seurat. Each of these clusters is then divided into patient-specific sub-clusters in a second step. In the last step, each of these patient-specific clusters is again subdivided by a refinement k-means clustering, where each cluster centroid forms a so-called meta-cell as a proxy for all single cells in the cluster (see **Figure 1** and **Supplementary Material** for details of the downsampling approach).

Downsampling reduces the number of single cells, and thus the runtime and memory usage of the SOM training by a factor of about 100. Importantly, all relevant expression patterns of the single cells are preserved in the meta-cell data after downsampling allowing for single-cell resolved downstream analyses. For example, myeloid cells make up only 0.2% of all cells, but their signature genes form a distinctive module as shown below.

### Self-organizing map and portraying of expression landscapes

SOM machine learning was then applied to meta-cells using mean expression values averaged over the single cells of each meta-cell, respectively. The SOM algorithm realizes two main tasks (26): Dimension of the single gene expression profiles was reduced into a set of meta-gene profiles by clustering similar gene profiles, followed by multi-dimensional scaling by mapping of each gene into the two-dimensional SOM grid. We used a parallelized SOM training algorithm implemented in Bioconductor R-package ‘oposSOM’ (16). Further information about the SOM training can be found in the **Supplementary Material** and the references therein.

Processing of expression data using the SOM method allows for comprehensive structuring and intuitive visualization of transcriptome landscapes (15). It translates meta-gene expression data into so-called expression portraits by aligning the meta-genes in a square grid according to the SOM’s topology and appropriate color-coding: We use a color gradient from dark red (meta-genes over-expressed in the respective sample(s)) to green (non-differential) to blue colors (under-expressed meta-genes).

These portraits serve as fingerprint of transcriptional activity of a meta-cell, a single cell or a subpopulation of cells (see below). Notably, expression information of all individual genes in the data set is represented in the portraits due to dimension reduction. Different expression portraits can be directly compared as the mapping of the genes to the meta-genes is fixed at the same position in all portraits.

### Expression modules and functional annotation

SOM machine learning arranges meta-genes with similar expression profiles in neighboring positions in the two-dimensional grid, dissimilar ones are located more distantly. In consequence, the expression portraits show smooth color textures with red and blue spot-like regions of correlated meta-genes concertedly over- and under-expressed, respectively. These clusters of meta-genes, in turn, are associated to genes differentially co-expressed in different cell types and states.

Data modularization is realized by clustering of meta-genes into disjoint modules which combine into patterns of activated modules (see below). Several modularization algorithms are implemented in oposSOM (15, 16, 38). In this publication we use the over-expression metric to define the expression modules: Accordingly, meta-genes exceeding a given expression threshold (here: 90% of maximum expression) were selected in each of the portraits. Then, spot module clusters were extracted as adjacent areas of the selected meta-genes. This approach ensures robust extraction of differentially co-expressed gene clusters (15, 17–20, 24, 31, 39). The modules encompass potential marker genes for the cell types showing specific overexpression of the respective spot module. Importantly, the algorithm determines the modules in an unsupervised fashion: Their number and co-expression combinatorics are an intrinsic measure of the complexity of expression patterns observed in the data.

The genes assigned to a particular expression module are assumed to share a common functional background (40). For functional annotation, we applied gene set enrichment analysis based on a collection of more than 8,000 gene sets derived from GeneOntology (41), GSEA (42), and KEGG (43) databases. Right-tailed Fisher’s exact test was used in each module to determine over-representation of set genes (38). Resulting top-enriched gene sets provide a data-driven view on the functional context of each of the modules.

As an option for hypothesis-driven functional mining, functional gene sets such as the T cell subpopulation markers are mapped into the SOM grid. Their localization in or near a spot module may imply functional association.

### Upscaling of meta-cell to single-cell data

Due to the vast number of single cells in the data set, a downsampling and refinement clustering were necessary prior to the SOM training. The obtained meta-gene expression data refers to the meta-cells instead of the single cells. For back-transformation into the higher granular single-cell level we applied support-vector machine- (SVM-) based prediction model to generate meta-gene expression values from the associated genes [see (44) and **Supplementary Material**]. This transfer learning predicts the 1,600 meta-gene expression values of each single cell in order to generate their expression portraits without the necessity to re-run the SOM algorithm. Module data is calculated for each single cell as the mean expression averaged over all corresponding module genes.

### Module activation patterns

Analysis of single-cell data provides eleven spot modules labelled with letters A- K. The most frequent modules are B and C, activated in 38% and 20% of all cells, respectively (see below). Rarest modules are F and K activated in 0.1% and 1.1% of the cells only (see **Supplementary Table S2**). Each cell can show either a single activated spot module, or combinations of them defining the particular module activation pattern (PAT) of the respective cell (17, 24). PATs are labelled using letters assigning the activated modules. For example, PAT ‘B C E’ denotes a cell with modules B, C, and E activated. In these labels, the module letters are ordered according to overall activation frequency of the modules across all cells, with the first letters referring to more abundant modules and the later letters to rarely activated modules.

The activation state of a module is determined by its expression value in a particular cell in comparison to the standard deviation of all module expression value in all cells. To generate PAT labels also for cells with less pronounced differential expression, we define so-called major and minor PATs, depending on the threshold applied: A cell assigned to a major PAT shows expression values of all activated modules exceeding one standard deviation of all module expression values. A minor PAT implies that all activated modules exceed a threshold of 0.5 x standard deviation with however at least one of the modules falling below one standard deviation.

PATs found in less than 0.5% of all cells (=667 cells) were rejected from further analysis to focus on recurrent patterns. A cell with no activated expression modules is assigned to ‘none’ PAT. In total, 46,010 cells (34%) were classified into 39 major PATs, another 73,777 cells (55%) into the corresponding minor PATs, and 13,618 cells (10%) remain unclassified (‘none’ PAT).

Hence, a PAT subsumes a group of cells showing a similar expression pattern, which, in turn, can distribute over different cell populations. Enrichment of PATs in the different cell subtypes (e.g., T cell subpopulations, ICANS groups) was calculated using Fisher’s exact test based on the PAT frequencies among the cells in the sets. By comparing the PAT frequencies of two cell subpopulations, we extracted a virtual ‘PAT flow’. The algorithm iteratively balances over-represented PATs in one part of the cells and most similar under-represented PATs in the other, and uses the differential frequencies to create a flow graph. An example is given in the **Supplementary Material**.

## Results

### Decomposition of cells into functional subpopulations

**Figure 2:**
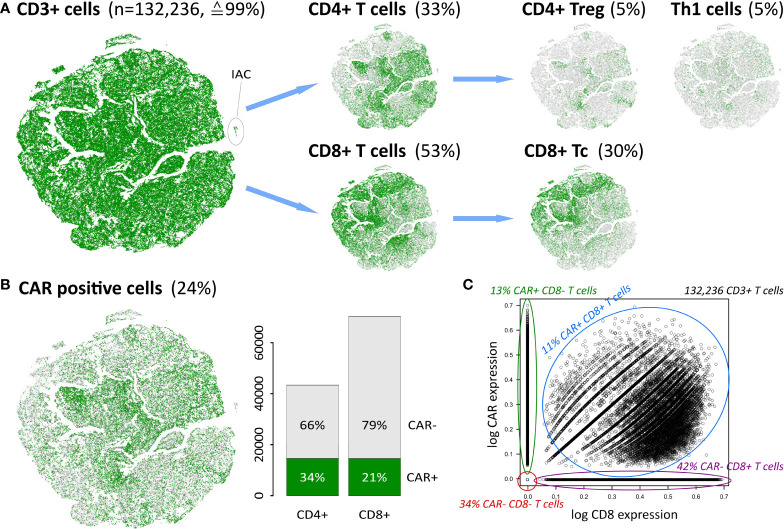
Cellular identity decomposition using consensus markers: **(A)** Assignment of T cell subpopulations and t-SNE projection. Percentages refer to total number of cells in the data. **(B)** CAR positive cells decompose into CAR+ CD4 and CD8 cells, respectively. A small population of ICANS-associated cells (IAC) with myeloid characteristics (14) segregate in a separate cluster as indicated. **(C)** CAR expression as a function of CD8 expression in CD3+ T cells reveals that double positive cells express both markers, while single positive and negative populations distribute along the y- and x-axis, respectively. Proportions given in the figure relate to the total number of 132,236 CD3+ T cells in the data set.

The infusion product of anti-CD19 CAR-T cell therapy comprises a variety of different T cell subpopulations, alongside a relatively small fraction of myeloid cells (14, 45). According to our classification scheme, T cells are defined as CD3 expressing (CD3+) cells and make up 99% of the total number of 133,405 cells (**Figure 2A**). Of those, only less than 0.5% are in a naïve state (**Supplementary Table S3**). About 0.2% of all cells are assigned as myeloid cells, expressing CD33 or CD11B instead of CD3, another 0.8% of the cells do not express any of the common immune cell markers CD3, CD19, or CD33. These results are in line with the targeted infusion quality (46), and serves as an option of molecular monitoring for quality assurance.

T cells are subdivided into CD4+ and CD8+ subpopulations, and further into the functional states naïve, memory, regulatory, helper, and cytotoxic T cells (**Figure 2A**). Th1, CD4+ Treg and CD8+ Tc cells thereby constitute the most frequent states and will be addressed in subsequent analysis steps. Naïve and memory cell subpopulations not shown in **Figure 2A** comprise less than 5% of all cells (see **Supplementary Table S3**).

Next, we investigated the number of CAR-positive (CAR+) cells as defined by the expression of the FMC63-CD19scFV marker (14). We found an overall proportion of about 24% of all cells **(** **Figure 2B**), which is in line with previous results (14). This percentage differs slightly between the CD4+ and CD8+ cells (34% and 21%, respectively), reflecting varying efficiency of the CAR gene transfer in these subpopulations (46, 47). About 8,000 cells (≙6%) are found to be double-positive CD4+CD8+. In total, 29,215 cells are either CAR+ CD4+ or CAR+ CD8+ (**Figure 2C** and **Supplementary Figure S6**).

### Data portraying and modularization of the single-cell transcriptomes

**Figure 3:**
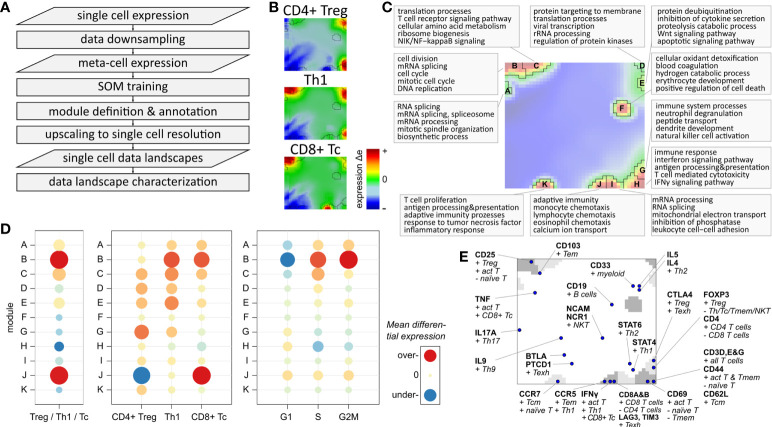
Expression landscape of T cells: **(A)** Flow chart of single-cell data portraying workflow (see **Supplementary Figure S5** for more details). **(B)** Expression portraits of most abundant subpopulations. Red and blue pixels represent meta-genes over- and under-expressed in the respective subpopulation, respectively, meta-genes colored in green show no differential expression. **(C)** Module definition and functional annotation. **(D)** Mean module differential expression averaged over all cells in the three main subpopulations, and grouped by subpopulation and by cell cycle phase, respectively. **(E)** Mapping of T cell and immune cell subpopulation makers.

We adapted our data portraying workflow developed for bulk transcriptomics to single-cell RNAseq data, which enables application of a series of downstream analysis tasks such as gene clustering and mapping into SOM space, visualization options, data modularization, function mining, and diversity analyses (15, 16, 27, 38). We supplemented the workflow with downsampling and upscaling steps to handle the large number of cells (**Figure 3A**). Their expression landscape portraits serve as fingerprint of the transcriptional state of the respective cell types which, in turn, are characterized by clusters of concertedly upregulated genes seen as colored spot-like areas (**Figure 3B**; red-to-blue refers to high-to-low expression, respectively): For example, CD4+ Treg and Th1 share common over-expression of genes located in the top-right and bottom-right corners of the portraits, with Th1 cells additionally showing over-expression of genes in the top-left corner. Notably, these genes are also over-expressed in the CD8+ Tc cells, together with CD8 specific genes located at the bottom edge of the map. Please note that here and in subsequent analyses the three subpopulations are restricted to mono-functional cells, i.e. cells that are assigned to only one functional state as defined by the marker genes. The characteristic spot-like patterns of the expression portraits segment into prominent regions of co-over-expressed (red spots) and co-under-expressed genes (blue spots). Such genes sharing a common mode of expression embody the so-called expression modules, clusters of co-regulated genes with coherent functional background (38, 48). The number of expression modules observed in the portraits characterizes the intrinsic diversity of the underlying transcriptional state space (15). The module map summarizes the global spot patterns, showing 11 modules labelled with capital letters A – K (**Figure 3C**). The functional context of spots A- C located in the left upper corner associates with cell cycle and proliferative activity including mitotic organization, DNA replication, cell division and biogenesis of cellular components (**Figure 3C**; top-5 enriched biological processes are shown for each module). Modules G – K, located on the bottom edge, enrich genes related to immune response, mainly responsible for adaptive immunity, inflammation, leucocyte proliferation and chemotaxis. Note that each single gene (as represented by individual Ensembl-IDs) can be assigned to only one module. Overlapping functions shared by multiple modules consequently refer to subsets of the utilized gene ontology signature (41), showing distinct modes of differential expression.

### Expression modules characterize specific functions of T cells

The modules differ strongly in their absolute expression levels averaged over all T cells (**Supplementary Figure S8**) and in their differential expression level between the three main subpopulations (**Figure 3D**): Overexpression of modules B and J is observed in Th1 cells (module B) and in CD8+ Tc cells (B and J). Module D shows by far highest overall expression level in all T cells, which can be therefore considered as T cell housekeeping module (see **Supplementary Figure S8**). The Tc specific spot module J is characteristic for CD8+ cells while CD4+ Treg and Th1 show similar patterns of modules C – I. The cell cycle associated module B is, on average, over-expressed in Th1 and Tc cells, which is mainly caused by a higher fraction of cycling cells among them (see below). This result is supported by the differential module expression grouped by cell cycle phases **(** **Figure 3D**, right part). It clearly shows activation of module B in the G2M and S phases. Modules A and C can be seen as a weaker signature of cycling cells in G2M (both modules) and S phases (module C only), while module H is over-expressed in G1 phase resting cells.

Complementary to the functional enrichment analysis, we projected common immune cell markers into the map (**Figure 3E**). Genes coding for CD69 and the CD3 delta, epsilon and gamma chains are contained in module H altogether, characterizing baseline T cell functionality. CD25, a common T cell activation marker, is located in module C, which is therefore considered as a co-signature of cell cycling together with module B. Markers of CD8 alpha and beta are found in module J together with IFNγ, PRF1, GZMA, and GZMB, which makes it the signature module for all CD8+ T cells. In general, we find a marked asymmetry of the distribution of these markers over the map: Only three of them are located in the top-left region of the map referring to cell cycle activity, while the other markers mainly distribute in the right lower region in or near the modules F – K, referring to different immune response mechanisms. Immune checkpoint inhibitors such as LAG3 and CTL4 associating with T cell exhaustion and ICANS are found in modules J and G, respectively (see below). CD8 T cell exhaustion was previously found to associate with poor treatment response and high ICANS in immune therapies of lymphomas (49). Genes of another ICANS-related signature (ICANS associated cells, ‘IAC’ (14)) locate in module F (IL1B) and K (SIRPA, LILRB4, CD68).

In summary, SOM portraying characterizes the transcriptomic states of the different T cells in terms of functional modules (**Table 1**). They can be understood as combinatorial building blocks of cellular functions including cell cycle and immune response potentially related to ICANS.

**Table 1:** Characterization of the expression modules.

Module	Major characteristics	Cell cycle	Cell type ^a^	Key genes in/near module
**A**	Cycling T cell signature	cycling cells (G2M & S phases)	CD4+ & CD8+ effector cells (≈20-25%)	CCNE2, CDC23
**B**	**Main cycling T cell signature**	**cycling cells** **(G2M & S phases)**	**CD4+ & CD8+ effector cells (≈30-35%)**	CD25, CD103 CCNA2, CCNB1, CCNB2, CDK1, CDK4
**C**	Cycling T cell signature (S phase)	cycling cells (S phase)	CD4+ & CD8+ effector cells (≈20-30%)	CCNH, MYC
**D**	High expression **T cell housekeeper** module	cycling cells (S phase)	CD4+ effector cells: Treg (≈20%), Th1 (≈20%), Th2 (≈40%), Th17 (≈55%), Th9 (≈80%)	SKP1, PSMA1, PSMB10, PSMD8, PSME1, FKBP1A
**E**	Co-activated with D, mainly in CD4+ cells	unspecific	CD4+ & CD8+ Treg (≈25%) Th1, Th2, Th9 & Th17 (≈25-35%)	CCND3, CDKN2A
**F**	Myeloid cell signature (rare)	unspecific	**Myeloid cells** **(≈85%)**	**CD33**, CD11C, CD163 CCNA1, CCND1
**G**	**Treg & Th** signature module	resting cells (G1 phase)	**CD4+ & CD8+ Treg (≈40-45%)** Th1 (≈30%), Th2 (≈40%)	**CD4**, FOXP1, CTLA4
**H**	Baseline signature of **G1 and resting CC cells**	**resting cells** **(G1 phase)**	All T cells (≈15-20%)	**CD3**, CD44, CD62L, CD69 CDC25B, CDKN1B
**I**	G1 & G2M signature	unspecific	CD4+ & CD8+ Treg & Th1 (≈15-20%)	CCND2, CDK6
**J**	**CD8** signature module	unspecific	**CD8+ Tem & Tc** (**≈45%)** CD8+ Treg (≈20%)	**CD8**, LAG3, TIM3, IFNγ, CCR5, PRF1, GZMA, GZMB
**K**	Myeloid cell signature (rare)	unspecific	**Myeloid cells** **(≈85%)**	CCR7, BTLA, PDCD1

a Percentages refer to the fraction of the cell type in relation to all cells expressing the module.Major characteristics and genes are highlighted in bold font.

### Combinatorial activation of expression modules deciphers T cell heterogeneity

**Figure 4:**
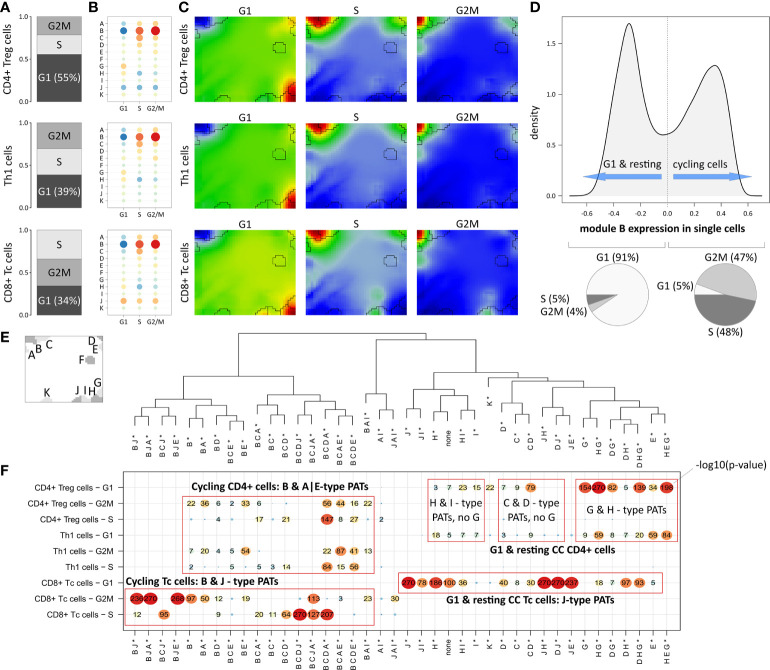
Segregation of Treg, Th1 and Tc subpopulations according to cell cycle phase: **(A)** Relative amounts of cells in G1, G2M and S phase, respectively. **(B)** Module expression grouped by subpopulations and cell cycle phase. Blue dots represent under-expressed modules, red dots over-expressed ones. **(C)** Corresponding expression portraits. **(D)** Distribution of module B expression in all cells shows bimodal character according to resting (G1-phase) and cycling (S- & G2M-phase) cells, respectively. **(E)** Module localizations in the map and hierarchical clustering of the PATs. * denotes that the PATs are not yet split into major and minor PATs. **(F)** The overview heatmap shows combinations of activated modules (PATs; major and minor PATs are summarized in this plot). Rows represent the subpopulations and cell cycle phases, columns represent the PATs clustered according to similarity of their average module expression. The numbers in the map show –log10 p-values derived from Fisher’s exact test. CD8+ cells show activation of module J related to LAG3-mediated T cell exhaustion partly together with cell cycle module B.

The expression landscape of T cell subpopulations as shown in **Figure 3A** can be understood as a superposition of expression patterns originating in the composition of the corresponding cells. Next, we aimed at decomposing T cell populations according to their cell cycle phase (i.e. cells in G2M or S; **Figure 4A**): About 55% of CD4+ Treg cells are assigned to G1 phase, meaning that the majority of this population are either cells with resting cell cycle or cycling cells momentarily in this phase. Note that the signatures utilized for cell cycle assignment do not distinguish between the G0 and G1 phases (37).

In Th1 and CD8+ Tc cells, the proportion of G1 cells is smaller (39% and 34%, respectively), which explains the observation that the main cell cycle signature (module B) is over-expressed in Th1 and CD8+ Tc cells in comparison to CD4+ Treg cells (**Figure 3B, D** **)**. The expression profile of module B expression is virtually identical in all three T cell subpopulations indicating their similar distribution over the cell cycle phases (see **Figure 4B** and the corresponding portraits in **Figure 4C**). Alike, the baseline G1 module H shows consistent expression patterns in the subpopulations. The distribution of module B expression supports the observation of its concerted over- and under-expression depending on the cell cycle status: The density plot of module B expression shows a bimodal distribution (**Figure 4D**), reflecting the split into cells currently in G1 phase (left peak) and cells in G2M or S phase (right peak, p<10^-16^ in Fisher’s exact test).

The expression patterns analyzed so far refer to mean values averaged over subpopulations of cells. To further decipher heterogeneity of single-cell states with finer granularity, we decomposed them into module activation patterns (PATs), which are defined by the combinatorial activation of modules on single-cell level. For example, PAT ‘B J’ denotes a cell with activated modules B and J (see Materials and methods section). A total number of 40 PATs can be distinguished (see dendrogram in **Figure 4E**). Enrichment analysis of the PATs reveal that CD8+ Tc cells associate to activated module J as a general feature (**Figure 4F**), which however splits into co-activations either with modules A, B and E, or with D, and H, depending on the cell cycle status. Cycling CD4+ cells reveal very similar enrichment of module B, A, and E related PATs, which however lack activation of J in contrast to the CD8+ Tc cells. G1 phase and resting CD4+ Treg cells differ from Th1 cells mainly by stronger enrichment of PATs containing modules G and H (see top-right part of the map in **Figure 4F**). PATs including modules G and J are found in resting CD4+ and CD8+ Tc cells, respectively, where activated modules J and G associate with immune cell exhaustion mediated by immune checkpoints CTLA4 (module G), and LAG3 and TIM1 (module J). These results demonstrate that PAT analysis disentangles the heterogeneity of cells of the same subpopulation into functional sub-states.

### The landscape of module activation patterns

**Figure 5:**
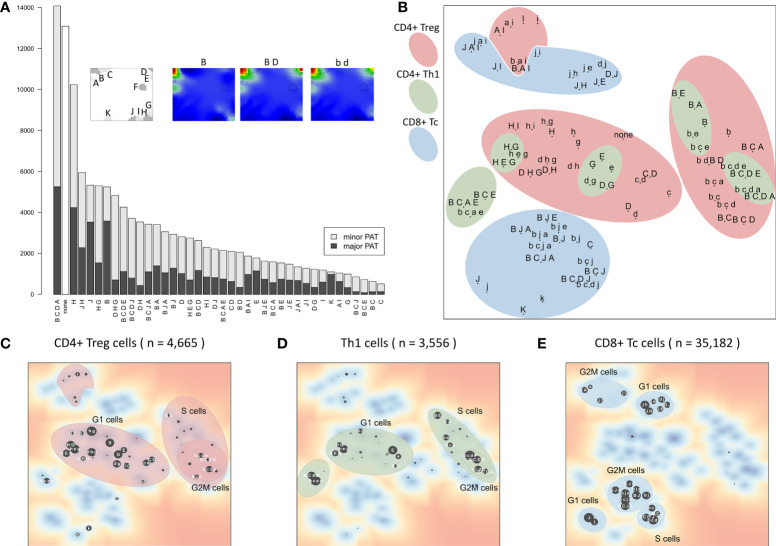
Module activation patterns (PATs) of activated expression modules reveal modular combinatorics: **(A)** Frequency of the module combinations detected in the cells. Major PATs collect samples with all modules exceeding the 1σ expression threshold, minor PATs those with all modules >0.5σ, but some <1σ (see also example portraits). **(B)** The PAT map is generated using t-SNE on PATs’ average expression values. PATs enriched in the three major T cell subpopulations and cell cycle phases are highlighted (see panels **(C–E)**. Red and blue areas include cycling (B-type) and LAG3-exhausted (J-type) PATs, respectively. **(C–E)** t-SNE map of PATs enriched in all CD4+ Treg, Th1 and CD8+ Tc, respectively. Size of the dots scales with enrichment (–log10 p-value in Fisher’s exact test, data is given as **Supplementary File 2**).

Activation of the modules show diverse subpopulation specific patterns. For a more detailed view, the 39 PATs (excluding the ‘none’ PAT) were further split into major and minor PATs according to a higher and lower stringency of the activation threshold applied, respectively. The example portraits in **Figure 5A** illustrate that the major PATs ‘B’ and ‘B D’ strongly express the respective modules, while minor PAT ‘b d’ resembles ‘B D’ however with module D on lower activation level. The major PATs comprise 34% of all single cells, minor PATs 55%, and another 10% remain unclassified (‘none’-PATs). The number of cells thereby varies over two orders of magnitude from several thousand cells in the most abundant PATs to few dozens in the infrequent ones (**Figure 5A**).

The t-SNE projection (36) visualizes similarity relations between the PATs and relates them to the respective cell types (**Figure 5B**). PATs, differing in only one or two modules, such as ‘H G’, ‘H E G’, and ‘D H G’, are located closely in the map as expected (central left part). Also, major and minor PATs of the same spot composition occupy close positions. The major/minor pair ‘B C E’/’b c e’ deviates from this rule owing to the enrichment of Th1 cells only in ‘b c e’ (p-value < 10^-5^ in Fisher’s exact test) in contrast to ‘B C E’ (p-value = 0.31), indicating different cellular origins of the respective expression patterns.

Interestingly, the PATs group into clusters of different cell cycle phases which differ for the three T cell subpopulations (**Figures 5C** **–E**). This agrees with our finding that expression of CD8+ Tc cells is distinct from CD4+ cells, which, in turn, show similar expression patterns in the Treg and Th1 subpopulations. It turned out that CD4+ Treg cells distribute over the widest variety of PATs, with Th1 related PATs as a subset (‘H D/G’ PATs for G1 cells, and ‘B C D A/E’ for cycling cells). This leads to the assumption that these PATs represent a basic CD4+ characteristic, and that the functional status of the cells modulates these patterns in agreement with the description of the individual modules (**Table 1**). In summary, the PAT map provides a reference coordinate system for the expression patterns observed in the different cells and subpopulations, which enables further analysis of their functional impact and mutual similarity.

### Decreased cell cycle activity relates to neurotoxicity

**Figure 6:**
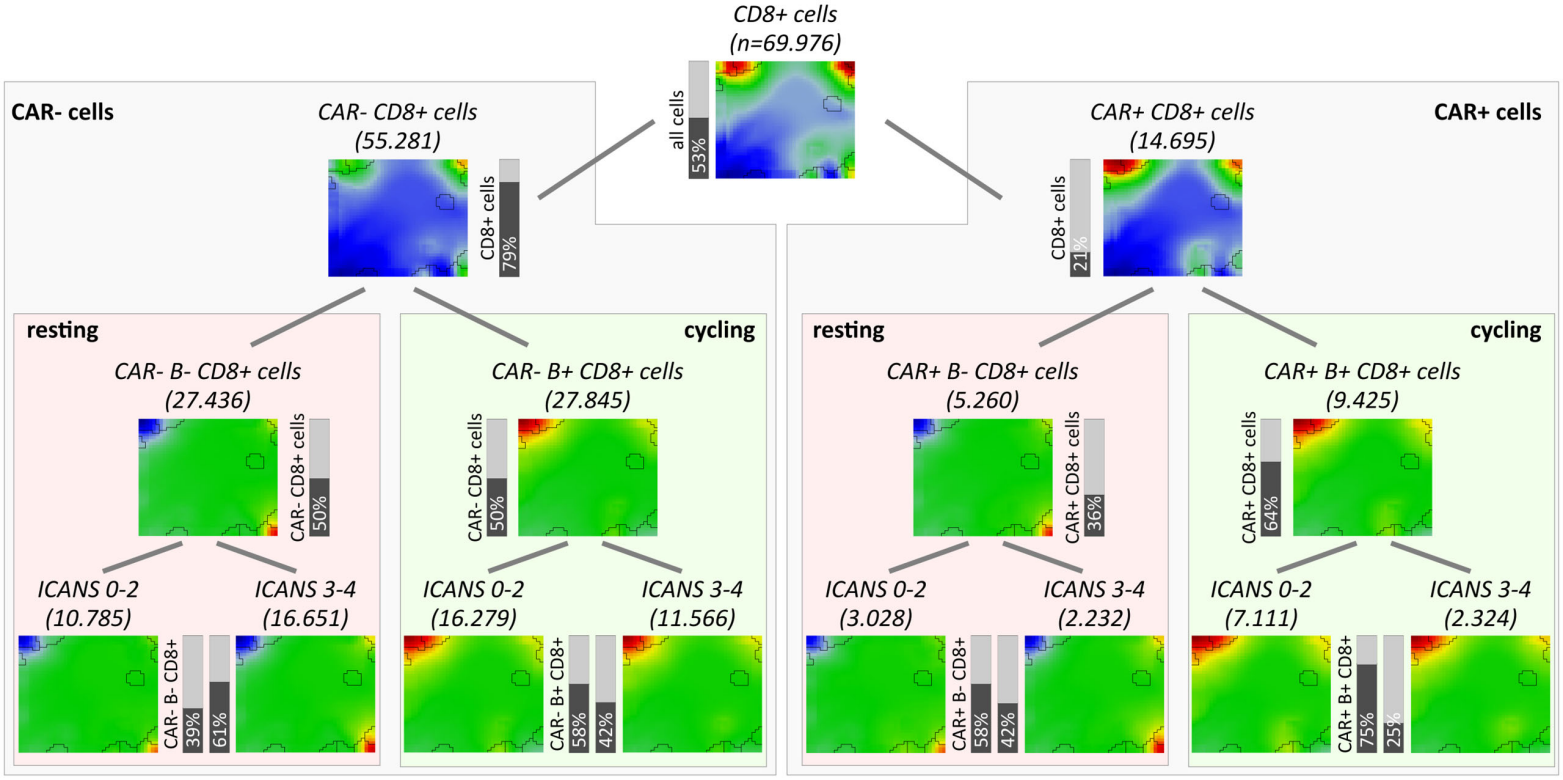
Overview of subpopulation expression portraits and relative abundances. CD8+ T cells were hierarchically stratified by CAR-status, by cell cycle activity as seen by module B expression, and by ICANS group (from top to bottom). Pairwise comparison of the ICANS portraits in the four main branches reveals virtually identical expression patterns between the low and high ICANS groups in each of the branches. Contrarily, the relative amounts of cells in the low and high ICANS groups show marked differences especially in cycling CAR-positive cells (right main branch) meaning that high ICANS associates with a roughly three times reduced fraction cycling CAR+ cells. This difference is reduced in non-cycling CAR+ and cycling CAR- cells and reverses in non-cycling CAR- cells.

As one goal of our analysis we aim at associating the fraction and functional background of immune effector cells with the ICANS status. For this, we investigated the relation between the degree of neurotoxicity as estimated by the ICANS grade and cellular characteristics of the infusion product by considering both the single-cell transcriptome landscapes and the respective cellular composition. For a first glimpse we calculated the mean expression portraits of CD8+ cells which were hierarchically subdivided by their CAR-status (CAR+ or CAR-), cell cycle activity using module B activation as proxy (module B+ or B-), and by their origin from low or high ICANS patient (grade 0-2 or 3-4, respectively; grading is shown in **Table S1** according to (14); **Figure 6**). The portraits are virtually indistinguishable between the ICANS low and high groups in the different strata which suggests similar expression properties. The number of CAR+ cells in the low ICANS group exceeds that in the high ICANS groups for resting (58% vs. 42%) and especially for cycling cells (75% vs. 25%). This result suggests an inverse association between ICANS and the amount of CAR+ cells, especially of cycling ones. Overall, we see an overlap of low ICANS and cycling cells, independent of the CAR status (data not shown). Note also that low ICANS scores associate with higher fractions of CAR+ cells (58% and 75% in resting and cycling cells, respectively) compared to CAR- cells (39% and 58%). Overall these results suggest that the composition and cell states of the CAR-T infusion affects neurotoxicity.

**Figure 7:**
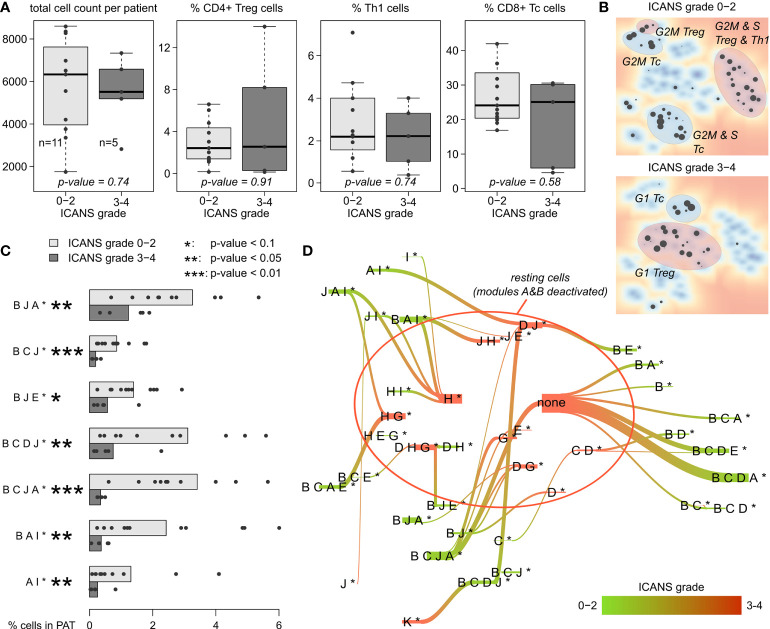
T cell subpopulations and expression patterns stratified by neurotoxicity grade: **(A)** Total cell numbers and relative amount of CD4+ Treg, Th1, and CD8+ Tc cells observed in male patients with ICANS grade 0-2 and 3-4, respectively. P-values were computed using Wilcoxon rank-sum test. **(B)** Maps of enriched PATs in cells grouped by ICANS grade. T cell subpopulations are highlighted according to **Figure 5**. **(C)** Fraction of cells in each male patient classified into the particular PATs (only PATs with p-value <0.1 in Wilcoxon rank-sum test are shown). Bar lengths represent the mean percentage for the two ICANS groups, the dots represent the individual patients. **(D)** Virtual PAT flow between ICANS groups. PATs on the green colored end of the flow are more frequent in ICANS grade 0-2 patient cells, those on the red colored end in ICANS grade 3-4. Width of the flow bars scale with the virtual flow.

For a more quantitative evaluation, we compared the total number of cells derived per patient between low (grade 0-2) and high ICANS (grade 3-4) levels (**Figure 7A**). We here restrict this analysis to men only, as there is only one woman in the low ICANS group. The total cell count is insignificantly higher in the low ICANS group (p-value=0.74; Wilcoxon rank-sum test). Also, the proportions of CD4+ Treg, Th1, and CD8+ Tc cells are similar when comparing the groups (p-values > 0.5; **Figure 7A**). None of the T cell subpopulations studied shows significant difference, indicating that the cell composition of the infusion products is virtually indifferent between the ICANS groups. Also, the total number of T cells sequenced per patient is not associated to ICANS (p=0.51 in linear model; see **Supplementary Figure S4** for T cell numbers). Please note that the total number of cells delivered to the individual patients is not known. Note also in this context, that there is also no significant difference between the infusion products of female and male patients in terms of cell number, subpopulations and proportion of CAR+ cells which implies extension of this result also to female patients (see **Supplementary Figure S10**).

Enrichment analysis of low and high ICANS associated PATs shows a clear difference between these groups in the PAT map, namely accumulation of the latter PATs more in the center and of the former ones closer to the edges of the map, which suggests differences in cell cycle activity (**Figure 7B**, compare with **Figures 5C** **–E**). Indeed, the high ICANS related PATs enrich G1 cells while the low ICANS related PATs associate with cycling CD4+ Treg and cycling CD8+ Tc cells in S and G2M phase. This result is supported by frequency analysis, providing seven differently populated PATs (p-values < 0.1, Wilcoxon rank-sum test; **Figure 7C**), all of them more frequent in the low ICANS group and six of them containing the cell cycle signature module B. The PATs reflect a dynamic cell state landscape with mutual transitions between them. The population flow diagram of PATs between the low and high ICANS groups further supports the association between cycling and low ICANS characteristics (**Figure 7D**): Cells derived from low ICANS patients mainly accumulate module B related PATs, which are mainly missing in the high ICANS group.

### Decreased CAR T+ content relates to neurotoxicity

**Figure 8:**
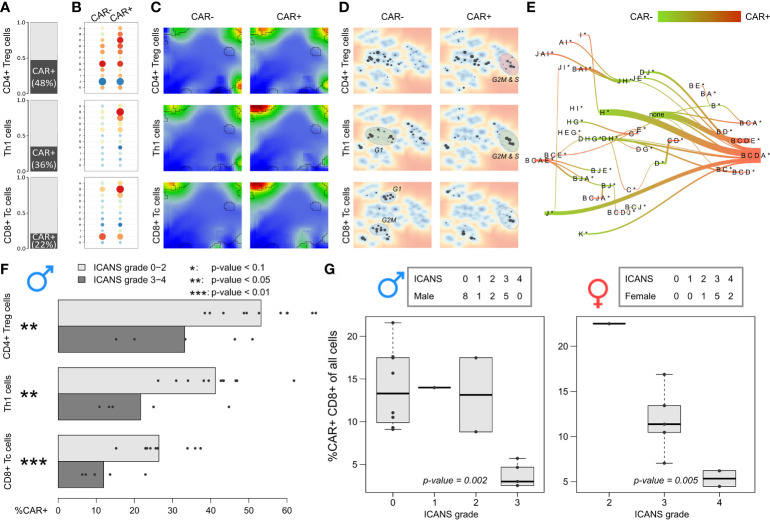
Expression patterns and relative abundance of CAR-positive and –negative T cells: **(A)** Relative amount of CAR+ cells in the subpopulations, respectively. **(B)** Module expression values grouped by subpopulations and CAR status. Blue dots represent under-expressed modules, red dots over-expressed ones. **(C)** Corresponding expression portraits. **(D)** Maps of enriched PATs in CAR-positive and –negative subpopulations. **(E)** PAT flow between CAR-positive and –negative cells. **(F)** Fraction of CAR-positive cells in each male patient grouped by T cell subpopulation. Bar lengths represent the mean percentage for the two ICANS groups, the dots represent the individual patients. p-values were computed using Wilcoxon rank-sum test. **(G)** Relative amount of CAR-positive CD8+ T cells in male (left frame) and female patients (right frame) grouped by ICANS grade. p-values were derived from linear regression model.

Our results indicated that the cells derived from high grade ICANS patients show decreased cycling activity as mirrored in the decreased amount of cycling cells in the respective infusion product. Next, we ask if the difference in cycling activity between the ICANS groups associates with the fraction of CAR+ cells in the subpopulations. It turned out that this fraction markedly differs between the three main subpopulations (**Figure 8A**): Almost half of the CD4+ Treg cells express the CAR marker, however only a third of the Th1 cells, and less than a quarter of the CD8+ Tc cells. These different numbers are comparable under the assumption that ‘false’ CAR- cells uniformly distribute over the subpopulations and indicate differing efficiency of CAR gene transfer as reported previously (46, 47). When comparing CAR+ and CAR- cells, over-expression of module B in CAR+ cells is conserved in all subpopulations, but also co-activation of signature modules A and C are in line with this trend (all p-values < 10^-15^, Wilcoxon rank-sum test; see **Figure 8B**). In support of this view, CAR+ cells are over-proportionally found in G2M and S phase (p-value < 10^-15^, Fisher’s exact test), indicating that these cells are predominantly in a cycling state. The corresponding expression portraits reveal module B as main distinctive feature, however with subtle subpopulation-specifics especially around CD8 module J in the bottom right part of the map, which is related to LAG3-mediated T cell exhaustion (**Figure 8C**). Overall, we find that cell cycle activity, exhaustion of immune checkpoint inhibitors (LAG3), low ICANS, and CAR+ state are correlated in part of the cells (**Supplementary Figure S11**). Hence, the CAR+ cells enrich in PATs associated to G2M and S phase of the cell cycle (**Figure 8D**), and in PATs involving module B and partly also J related to exhaustion in general (**Figure 8E**).

The fractions of CAR+ cells in the low ICANS group (grade 0-2) in the three main subpopulations clearly exceeds that in the high grade 3-4 group (p-values < 0.05, Wilcoxon rank-sum test; **Figure 8F**). This is in line to the previous observation that the overall percentage of CAR+ cells is higher in low ICANS patients (14). Note here, that two patients of the high ICANS group show very few CAR+ cells (<5 Th1 and CD4+ Treg). We extended this approach to the other T cell subpopulations by using linear regression to model ICANS grade as a function of CAR+ fractions in the subpopulations separated by the sexes and also for all patients taken together (**Supplementary Table S4**). It turned out, that the association of higher CAR+ fractions and lower ICANS grade can be found in several T cell subpopulations, with strongest effects for CD4+ cells (especially CD4+ Treg and Th2 cells; p-values < 0.01 for models including all patients). The relative amount of CD8+ Tc cells in relation to all cells derived from a patient significantly associates to ICANS grade (**Figure 8G**). Particularly, it markedly decays in the highest ICANS grades 3 and 4 in women and men. These results clearly show that the patients show ICANS-specific proportions of CAR+ cells. Note that the total cell number sequenced per patient is not associated with ICANS grade (p-value = 0.52 in linear regression model).

In summary, the degree of ICANS seems not to be associated with differing composition of the patient’s infusion product with regard to the T cell subpopulations studied. Instead, cells derived from high grade ICANS patients show decreased cycling activity and contain a decaying amount of CAR+ cells, which both associate with neurotoxicity.

## Discussion

### scSOM data portraying deciphers the diversity of transcriptomic states of CAR-T infusion products

We analyzed single-cell expression data of the infusion products of anti-CD19 CAR-T cell therapy provided by Deng et al. (14) by applying a newly-developed single-cell data portraying approach based on self-organizing map (SOM) machine learning (scSOM). The data comprise more than one hundred thousand individual cells derived from 24 patients, which was downscaled by about two orders of magnitude for effective training of the SOM. Therefore, we generated meta-cells as representative proxies of transcriptional states, which were again upscaled after SOM training to cover the whole diversity of transcriptomic state space with single-cell resolution.

**Figure 9:**
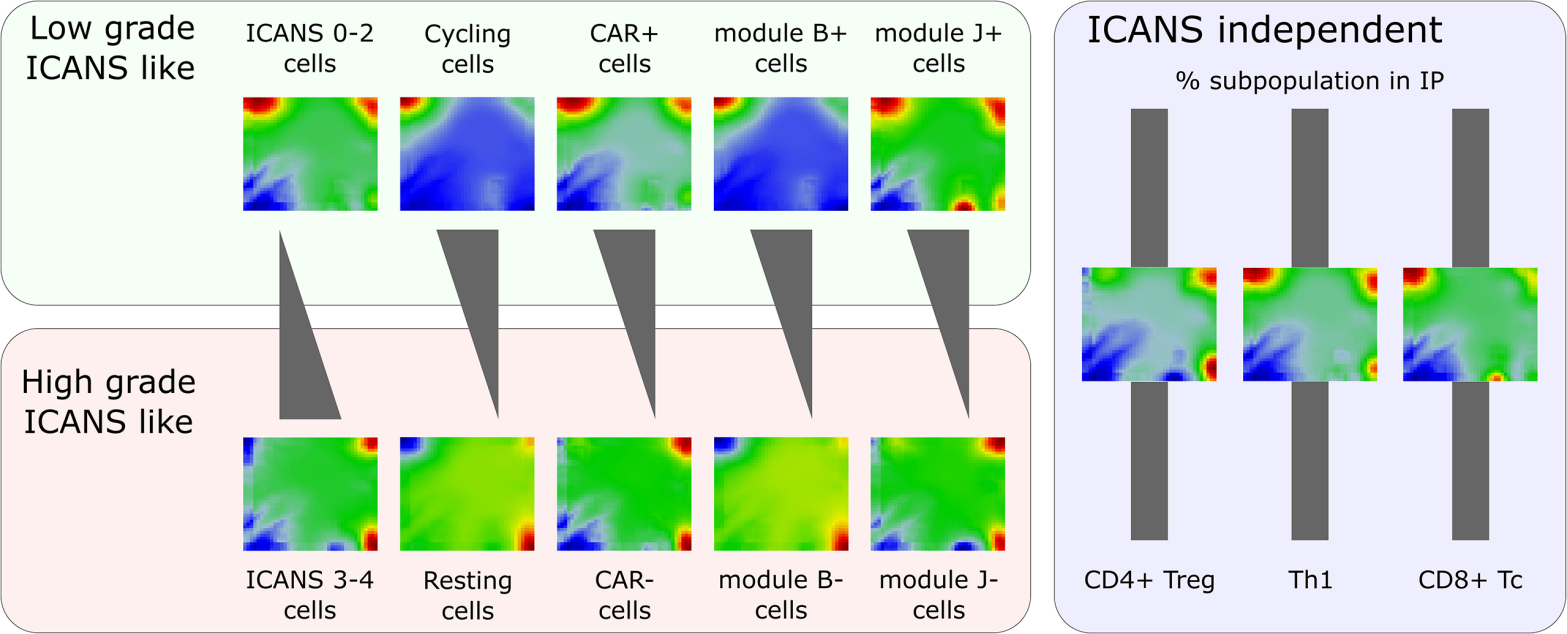
Portraying of single-cell transcriptome landscapes illustrates that neurotoxicity associates with decreasing cycling activity, amount of CAR+ cells, and expression of modules B (cell cycle genes) and J (exhaustion related genes LAG3 and TIM3). The composition of the infusion product (IP) regarding different T cell types is virtually invariant. The figure shows mean expression portraits of all single T cells stratified by ICANS group, cell cycle phase (S & G2M versus G1), CAR status, and expression of modules B and J, respectively.

The scSOM data portraying visualizes the whole transcriptome expression patterns of different cell types and states for the ICANS-low and ICANS-high groups, thus providing an intuitive view on differences which possibly associate with treatment adverse effects (**Figure 9**). The novel method identified 11 modules of co-regulated genes in the single-cell transcriptomes forming combinatorial building blocks of cellular functions in the different cell populations with implications to ICANS: Part of the modules relates to cell cycle activity, another part is T cell subtype specific. Interestingly, only one T cell subpopulation marker was found in the cell cycle related modules, namely CD25 (IL2RA), presumably due to the general role of CD25 in activated T cells, which also show enhanced proliferative activity. The other T-cell markers mainly distribute in or near the modules related to immune response mechanisms in a T cell subtype-specific fashion, as expected.

Another methodical novelty is the utilization of patterns of activated modules to stratify the single-cells into so-called PATs, representing unique states of upregulated transcriptional programs of the cells. This unsupervised, data driven clustering of cells provides higher granularity of cellular states compared to the Seurat clusters often utilized for downstream analyses. The PAT analysis disentangles the heterogeneity of cells of the same subpopulation into functional sub-states. PAT maps provide a reference coordinate system for the expression patterns observed in the different cells and subpopulations.

Importantly, the expression landscape established here will be used as a reference system to map independent data, for example to investigate expression patterns in published single-cell RNAseq data [e.g. derived from (13) or (50)], to disentangle cellular identities and transcriptional states of bulk samples, and to extend the analyses presented here by future studies in terms of additional patient samples and longitudinal measurements. scSOM provides two different approaches for this: Firstly, novel data can be projected into the map, providing expression portraits, module expression patterns, and PAT assignments for direct comparison with the results discussed here. Secondly, the sets of genes, which constitute the modules, can be exploited in gene set enrichment analyses and for generation of gene set maps in data portraying analyses of the new data. Such subsequent knowledge linkage is valuable and helps to understand transcriptional patterns in the new data, their functional background and their diversity, but potentially generates also new insights into the reference data.

Our study is important for quality assurance and control on the one hand, and for investigation of frequencies and mutual interactions between the subpopulations. We found an overall proportion of about 24% CAR positive (CAR+) cells, which is in the targeted range of transduced cells (46, 51). Cells in the CAR negative (CAR-) group (i.e. cells with no read found for the CAR marker FMC63-CD19scFV) potentially express this marker undetectably due to technical limitations of the sequencing process (14). Also dynamics of transcriptional regulation may disturb a clear coincidence of CAR gene transcription and CAR surface protein presence. An estimation shows that about 20% of CAR- cells show expression patterns similar to CAR+ cells (14), thus suggesting that part of CAR- cells are either false negatives or not distinguishable from CAR+ using transcriptional patterns, which in consequence weakens effects seen by analyses related to the nominal CAR- cell frequencies.

### Adverse neurotoxicity associates with increased contents of low-cycling, CAR negative and LAG3-exhausted cells

We find that neurotoxicity (ICANS) observed in the patients after CAR-T infusion does not depend on the T cell composition, but, instead, on differences in their functional state (**Figure 9**). Particularly, we did not find differences between abundances of T cell subpopulations taken from patients with low (0–2) and high (3-4) ICANS grade, suggesting that infusion product preparation preserves cellular composition and is not itself associated to adverse neurotoxicity. We have shown that the amount of cycling (G2M and S phase) cells is higher in low ICANS patients (59% vs. 43% in high ICANS; p-value=0.04). Moreover, the proportion of CAR+ cells is higher in patients with low grade ICANS. In other words, CAR+ cells associate with proliferative activity by unknown reasons (more than 60% of CAR+ cells are in cycling state, but less than 40% of the CAR- cells). One could hypothesize that a cellular state of proliferative activity promotes the CAR gene transfer. Data of the isolated T cells and of the derived infusion product would be valuable to investigate proportions of cycling T cells and subsequent CAR positivity in a series of patients, helping to understand if the physiologic state of the T cells affects CAR transfer efficiency and to exclude putative genetic biases. Interestingly, association between proliferative activity, checkpoint inhibition, long-time responsiveness to immunotherapy and self-renewal capacity related to T-cell exhaustion was recently reported suggesting a complex and only partly understood relation between cell-cycle and T-cell function (52).

CAR+ and CAR- cells markedly differ in their expression landscapes (**Figure 9**). Their relative amount is not only a quality measure of the infusion products, and increased fraction of activated CAR+ cells associates to less neurotoxicity: Linear regression analysis revealed significantly higher CAR+ fractions in lower ICANS grade patients throughout the different T cell subpopulations, especially in CD4+ cells, but also an increased relative amount of CAR+ CD8+ Tc cells in relation to all cells was found.

The expression landscape portraits for different T cell subsets summarize these findings (**Figure 9**): Mean portraits averaged over T cells derived from low ICANS patients clearly resemble those of cycling and CAR+ cells, as well as those of cells with activated modules B and J. As major characteristic, one finds overexpressed spots (red color) on the top edge of the portraits (modules B, C, and, partly, D). In contrast, portraits of high ICANS, resting, and CAR- cells, and cells with modules B and J inactive consistently show overexpression of modules G and H in the bottom-right corner of the map.

Overall, our results support the view that heterogeneity in the cellular and molecular features of CAR T cell infusion products contributes to variation in efficacy and toxicity after CAR T cell therapy in lymphomas (14). The absence of the FMC63 epitope in CAR- and/or cell cycle arrested cell states could potentially serve as quantifiable phenotypes which associate with neurotoxicity of the infusion product. The question, if these phenotypes are actionable by depleting them as undesirable, cellular populations or functional states during manufacturing, needs further studies.

In agreement with Deng et al. (14), we also find that LAG3-mediated T cell exhaustion (increased expression of module J including LAG3 and TIM3 genes) can be found in combination with cell cycle and CAR+ associated modules B and D especially in CD8+ Tc cells. Hence, the use of LAG3 and/or TIM3 blockade after infusion might improve efficacy and/or avoid neurotoxicity as previously proposed (14, 53). We have shown that the different subpopulations show very diverse module B vs. module J expression patterns, and, in particular, that myeloid cells associate high ICANS with resting and low LAG3-exhaustion (**Figure S11**). However, the exhaustion status of the immune cells before the CAR-T cell therapy is unknown and possibly influence these results.

Recently it was reported that mural cells, which surround the endothelium and which are critical for blood-brain-barrier integrity, express CD19, making them a possible on-target mechanism for CD19 CAR-T cell-mediated neurotoxicity, particularly because the CD19 isoform expressed in the adult brain contains the FMC63 epitope that is recognized by clinical-grade CD19 CAR-T cells (54). In this context, one could hypothesize that lower level of LAG3-mediated exhaustion associates with higher targeting and resulting neurotoxicity. It remains however unclear, why interaction of cycling CAR+ T cells with CD19 on endothelium leads to less severe ICANS than interaction of resting cells.

In addition to CAR-T cell-intrinsic effects, the clinical manifestation of severe neurotoxicity is a complex multifactorial process depending on lymphodepleting or chemotherapy regiment, scFV specificity and co-localization of on- and off-target cells [see discussion in (54)]. Also, immune monitoring will add valuable data to be considered in our analyses in future studies. Thus, our study can only add molecular details about the transcriptomic landscape with single cell resolution without being able to provide causal relationships usable to overcome neurotoxicity. SOM single cell portraying provides an option to study further details of the expression landscapes of the CAR-T infusion product to address these issues on molecular level.

## Conclusions

We provided a comprehensive single cell data portraying and demonstrated its capabilities to disentangle transcriptional states using intuitive visualization, functional mining, molecular cell stratification, and variability analyses. We have shown that scalable resolution from single-cell- to subpopulation-level generates novel insights into high-dimensional data sets, and particularly of CAR-T infusion products which extend the results reported in the original publication: We find that the transcriptional states of cells derived from patients with low ICANS grade do not differ from those of cells from high ICANS patients, while their relative abundancies with regard to cell cycle activity, CAR status and T cell exhaustion vary markedly.

Our results indicate that therapies can be improved by depletion of cell cycle arrested and CAR- cells, however CAR-T cell immunotherapy optimization needs further studies with higher number of patients involved to monitor a broader range of cellular and transcriptional states.

## Data availability statement

The single-cell transcriptome dataset analyzed for this study can be found in the Gene ExpressionOmnibus under accession number GSE151511 (https://www.ncbi.nlm.nih.gov/geo/query/acc.cgi?acc=GSE151511).

## Ethics statement

The studies involving human participants were reviewed and approved by Internal Review Board of MD Anderson Cancer Center (original publication by Deng et al.). The patients/participants provided their written informed consent to participate in this study.

## Author contributions

Conceived and performed analysis, major contributors to the manuscript: HL-W, HB. All authors contributed to the article and approved the submitted version.

## Funding

This publication is part of the imSAVAR project, which received funding from the Innovative Medicine Initiative 2 Joint Undertaking (JU) under grant agreement No 853988. The JU receives support from the European Union’s Horizon 2020 research and innovation programme and EFPIA and JDRF INTERNATIONAL. Publication was supported by the Open Access Publishing Fund of Leipzig University and the German Research Foundation.

## Conflict of interest

The authors declare that the research was conducted in the absence of any commercial or financial relationships that could be construed as a potential conflict of interest.

## Publisher’s note

All claims expressed in this article are solely those of the authors and do not necessarily represent those of their affiliated organizations, or those of the publisher, the editors and the reviewers. Any product that may be evaluated in this article, or claim that may be made by its manufacturer, is not guaranteed or endorsed by the publisher.

## Supplementary material

Supplementary File 1 Supplementary methods, figures and tables.

Supplementary File 2 PAT frequencies and significance levels grouped by mono-functional T cell subpopulations.

Supplementary Figure 1 Classification of T cell subpopulations using consensus markers.

Supplementary Figure 2
**(A)** The three downsampling steps.

Supplementary Figure 3 Illustration of the PAT flow algorithm.

Supplementary Figure 4 Proportion of CAR-positive cells in each of the participants with increasing ICANS grade.

Supplementary Figure 5 Flow chart of the CAR-T single cell data portraying workflow.

Supplementary Figure 6 CD4 and CAR expression characteristics in CD3+ T cells.

Supplementary Figure 7 Expression landscape portraits of the T cell subpopulations.

Supplementary Figure 8 Mean module overall expression level.

Supplementary Figure 9 Expression of module B in single cells.

Supplementary Figure 10 Comparison of infusion product composition and single cell expression patterns between female and male patients.

Supplementary Figure 11 LAG3-exhaustion score (module J) as a function of the cell-cycle score (module B).

Supplementary Table 1 Patient characteristics.

Supplementary Table 2 Number of genes associated to the expression modules and number of cells with module expression exceeding one standard deviation.

Supplementary Table 3 T cell subpopulations assigned using the consensus marker scheme. Poly-functional cells are counted multiply.

Supplementary Table 4 Regression analysis of ICANS grade.
