# Supplementary material for: Transcriptional states of CAR-T infusion relate to neurotoxicity – lessons from high-resolution single-cell SOM expression portraying
Source: Front Immunol. 2022 Sep 28;13:994885. doi: 10.3389/fimmu.2022.994885 (PMC9558919; doi:10.3389/fimmu.2022.994885)
Supplement: Supplementary File 1 — Supplementary methods, figures and tables. [file DataSheet_1.pdf]

Transcriptional states of CAR-T infusion relate to neurotoxicity – lessons from high-resolution single-cell SOM expression portraying

Henry Loeffler-Wirth, Michael Rade, Arsen Arakelyan, Markus Kreuz, Markus Loeffler, Ulrike Koehl, Kristin Reiche, Hans Binder

Supplementary Material

1 Supplementary Methods

1.1 Preprocessing of scRNA-Seq data

We applied Seurat R-package (1) for preprocessing of the read count matrix downloaded from Gene Expression Omnibus under accession number GSE150992. Parameters were chosen in accordance to the original publication (2).

i) Quality control & filtering: Firstly, cells with less than 200 genes and genes present in less than three cells were removed. Secondly, cells with more than 15% of the counts mapping to mitochondrial genome were removed too. Finally, cells with more than 7000 genes with non-zero count were also discarded.

ii) Normalization, feature selection & scaling: Log-normalization was applied using a factor of 10,000, followed by selection of variable features using variance-stabilizing transformation (vst). Then, linear data centering scaling was applied, resulting in genes' mean expression value of 0 with a variance of 1 in each gene.

iii) Dimension reduction and cell clustering: Seurat functionalities were applied to generate t-SNE (3) mapping of the cells and subsequent unsupervised cell clustering using 50 principal components. We here used more dimensions of the data as we intend to capture also smaller effects in downstream analyses.

1.2 T cell subpopulation markers

Marker genes for T cell populations were obtained from previously published signatures (2,4–9). Robust consensus markers were selected for major cell type and functional state assignment (Figure S 1). Essential marker genes are shown in green color and the corresponding read counts in a cell are required to be non-zero. Additionally, all absence makers (if any) are required to have no read counts. All T cell subpopulations share common presence of CD3.

Each cell in the data is assigned as CAR-positive or -negative, independent of cellular identity. To determine this CAR status, we used the CAR-specific sequence contigs48 (FMC63-CD19scFV, GenBank: HM852952.1) (2).

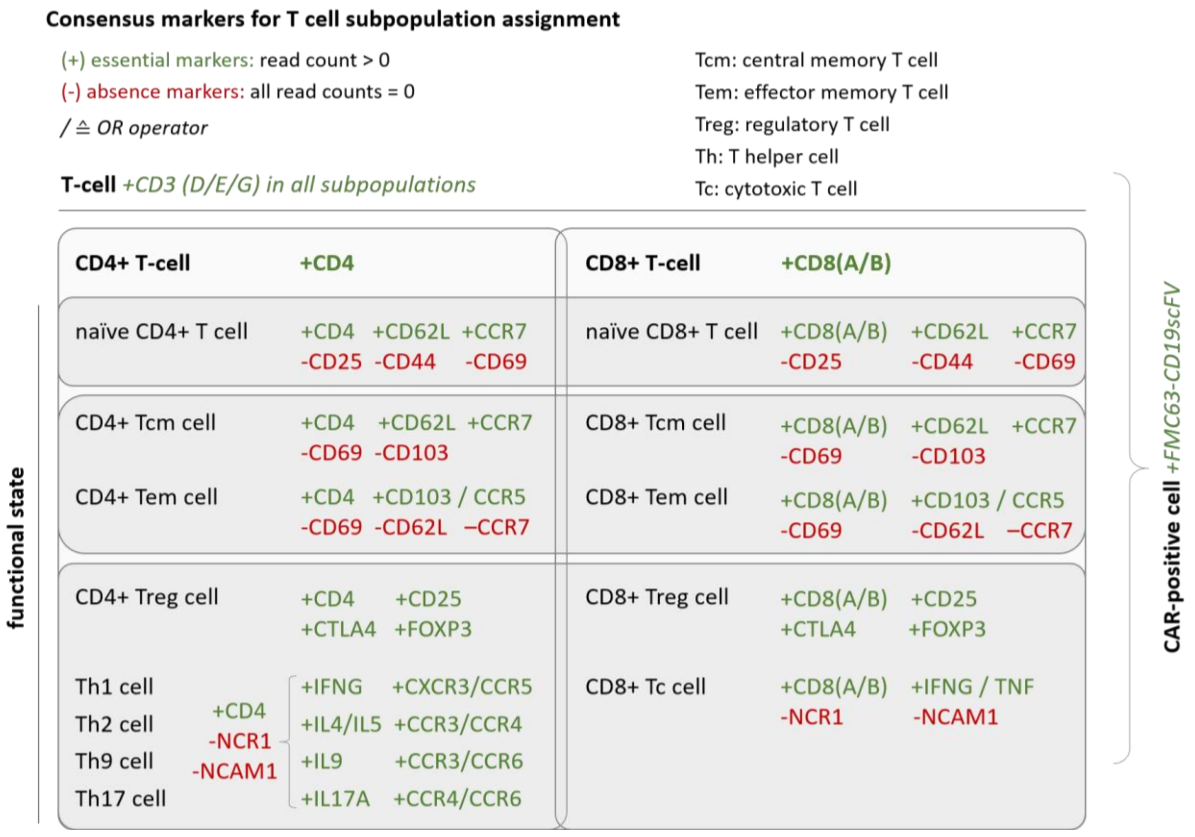

Figure S 1: Classification of T cell subpopulations using consensus markers. Marker genes in green color are essential with read count required to be non-zero, counts of absence markers in red color are required to be zero. Non-zero CD3 count applies to all T cell subpopulations.

1.3 Downsampling and refinement clustering of meta-cells

Generation of the meta-cells is a three-step downsampling algorithm (Figure S 2a):

i) Unsupervised clustering of all single cells is provided by the Seurat workflow. We obtained 30 Seurat clusters collecting between 2 and 15,684 cells (on average: 4,447 cells). We rejected 7 virtually patient-specific clusters, were more than 80% of the included cells originate from only 2 different patients, respectively. The removed clusters comprise in total 9,738 cells (=7.3% of all cells).

ii) The 30 clusters were then subdivided according to patient number, such that each sub-cluster includes cells from one particular patient and one particular Seurat cluster solely. In consequence, the resulting 523 clusters patient-specifically cover all 23 remaining Seurat clusters and all 24 patients.

We removed 63 sparse clusters with less than 10 single cells included, respectively, this way discarding 312 cells (=0.2%).

iii) The 460 clusters were subdivided depending of the number of single cells contained. For each cluster, k-means was applied to cluster the corresponding cells based on their expression data. The number of sub-clusters (centers) was thereby chosen depending on the number of cells (k = #cells / 100), resulting in more sub-clusters for higher numbers of cells. This quantitative sub-clustering finally leads to 1,486 clusters called meta-cells, containing 123,355 single cells (=92.3%) which ensures that all important expression patterns of the single cells are represented in the meta-cell data. On average, 83 single cells are contained in each meta-cell (Figure S 2b).

The 10,050 cells not covered by the meta-cells will not contribute to SOM training, however upscaling of module expression data, assignment of module activation patterns (PATs), and generation of expression landscape portraits are applied to them as well.

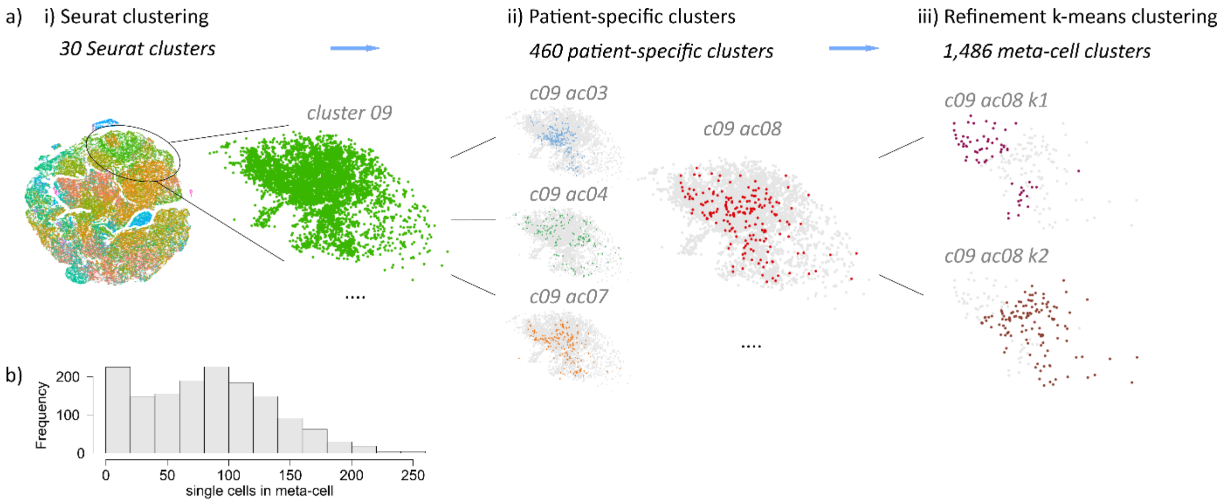

Figure S 2: a) The three downsampling steps: i) Seurat unsupervised clustering; ii) patient-specific subclusters; iii) refinement k-means clustering providing the meta-cell clusters. b) Distribution of the number of single cells contained in the meta-cell clusters.

1.4 Self-organizing map training

The self-organizing map (SOM) algorithm realizes three main analysis tasks (10):

i) Dimension reduction: The SOM is constituted of 1,600 so-called meta-gene entities arranged in a two-dimensional 40 x 40 grid. Each meta-gene represents a cluster of genes and is characterized by an average gene expression profile of the corresponding single-cells. The term 'profile' hereby denotes the vector of expression values across the meta-cells used as input data. In the iterative SOM training process, meta-gene profiles are gradually updated to optimize coverage of the data space as seen by the expression profiles of all genes. After the training algorithm converges, the original expression data matrix consisting of 20,629 gene profiles is transferred into a dimension reduced matrix of 1,600 meta-gene profiles.

ii) Clustering: For each gene profile, the best matching meta-gene profile is determined using Euclidian distance between the expression profiles as similarity measure. In turn, this gene to meta-gene association realizes a clustering of gene profiles to the corresponding meta-genes. The number of gene profiles collected in each individual meta-gene cluster thereby usually varies between only few to several hundreds of genes with very similar expression profiles.

iii) Multi-dimensional scaling: as the meta-genes are arranged in a square grid, the association of single genes to the corresponding best matching meta-gene implies a mapping of the genes into a discrete and non-linear two-dimensional space (10). Thereby the training algorithm ensures that genes with similar expression profiles cluster are mapped into the same or in closely located meta-genes.

The size of the map, i.e. the number of meta-genes, was chosen in accordance to recent studies (11–15) to allow robust definition of expression modules. These sets of meta-genes typically comprise dozens to few hundreds of single genes with similar expression profiles and concerted involvement in cellular functions.

We used a parallelized SOM training algorithm implemented in Bioconductor R-package 'oposSOM' (16).

1.5 Upscaling of meta-gene expression data to single cell level

The meta-gene expression matrix obtained from SOM training is based on the meta-cells used as training data. For the purpose of generating expression portraits of single cells and cell subpopulations, we use a SVM-based approach to compute meta-gene expression given the full set of single gene expression values for any interesting cell or subset (17).

Our approach uses 1,600 individual support-vector machines (SVMs) (18). Each one represents one meta-gene and is trained using single gene expression profiles along the meta-cells as independent variables and the corresponding meta-gene profile as depended variable. Thereby, only genes associated to the particular meta-gene or to one of the adjacent meta-genes are considered as predictors. After the SVM model is trained, it is capable to predict the 1,600 meta-gene expression values for any input of 20,649 (single gene) expression values. We used the expression values of a single cell, or the mean expression per gene averaged over all cells of a subpopulation to visualize the corresponding expression landscape portraits.

Note that the SVM upscaling is used only for the visualization of expression portraits. Single-cell module expression is directly calculated as the mean expression value averaged over all corresponding module genes.

1.6 Differential PAT enrichment and virtual PAT flow graph

Enrichment of PATs in a cell subset is calculated using Fisher's exact test based on the PAT frequencies. When two disjoint subsets are assessed, the difference of the PAT frequencies (percent of cells assigned to the PAT in the respective subset) can be further utilized to create a virtual flow graph under the assumption that cells in a PAT in the one subset correspond to cells with a similar PAT classification in the other subset. This graph visualizes mutually corresponding cell populations in the PAT map.

At first, the algorithm to create the flow graph initializes differential (d)-scores for each of the PATs as the difference of frequency proportion in the two subgroups (Figure S 3b). Then all branch mergings of the PAT hierarchical clustering dendrogram are visited with increasing height (major and minor PATs are summarized; see Figure S 3a). In each iteration, the algorithm evaluates if a flow between the respective two sub-branches is possible: This is the case if the corresponding sub-branch sums of d-scores have differing signs, i.e. if the PATs of one sub-branch are over-represented in one subgroup and the PATs of the other branch over-represented in the other subgroup (Figure S 3b). Then a flow is defined from positive to negative d-scores as the maximum absolute value of the sums of d-scores of the sub-branches, and the d-scores are accordingly updated: PATs with positive d-scores act as sources

of the flow (flow is subtracted from d-score), and the corresponding most similar PAT with negative d-score as sink (flow is added to d-score). The algorithm finally terminates with the merging of the two main branches, eventually balancing all d-scores to 0.

In result, the flow graph comprises the PATs as nodes and all flows as weighted edges. The graph layout (i.e. position of the nodes) is determined by the PAT map as provided by PAT expression tSNE analysis.

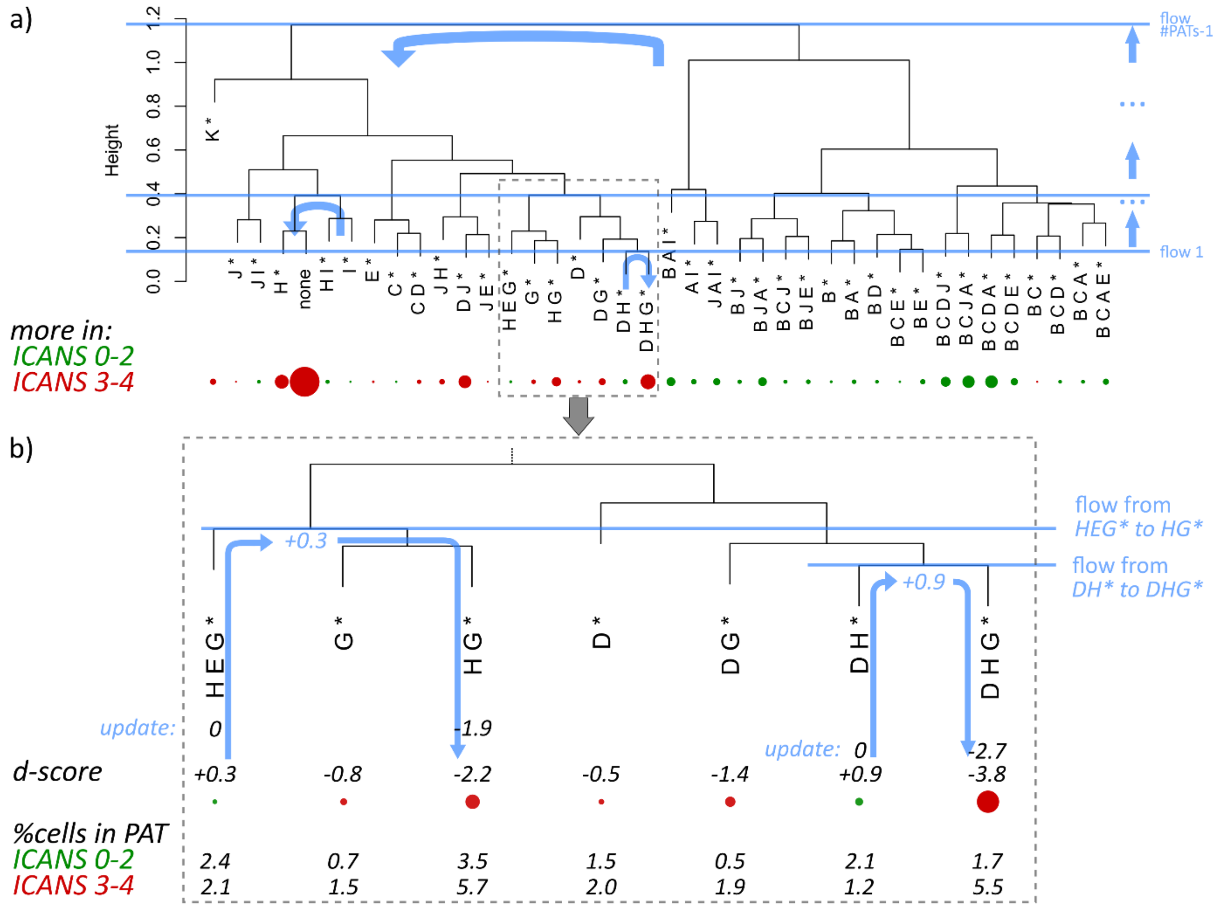

Figure S 3: Illustration of the PAT flow algorithm: a) The algorithm successively visits all mergings of the hierarchical clustering dendrogram with increasing height. The flow intends to balance over- and under-represented PATs in the corresponding sub-branches. b) Each leaf of the dendrogram obtains a d-score, which is initialized as the difference of frequency proportion in the ICANS 0-2 and 3-4 subgroups. A flow between any two sub-branches is possible, if the corresponding sums of d-scores has differing signs (e.g. merging of HEG* and G*/HG* with +0.3 and -3.0, respectively). Then a flow is calculated from positive to negative d-score (e.g. a flow of 0.3 from HEG* to HG*), adding the resulting flow to the flow graph and updating the respective d-scores prior to the next iteration.

2 Supplementary Figures

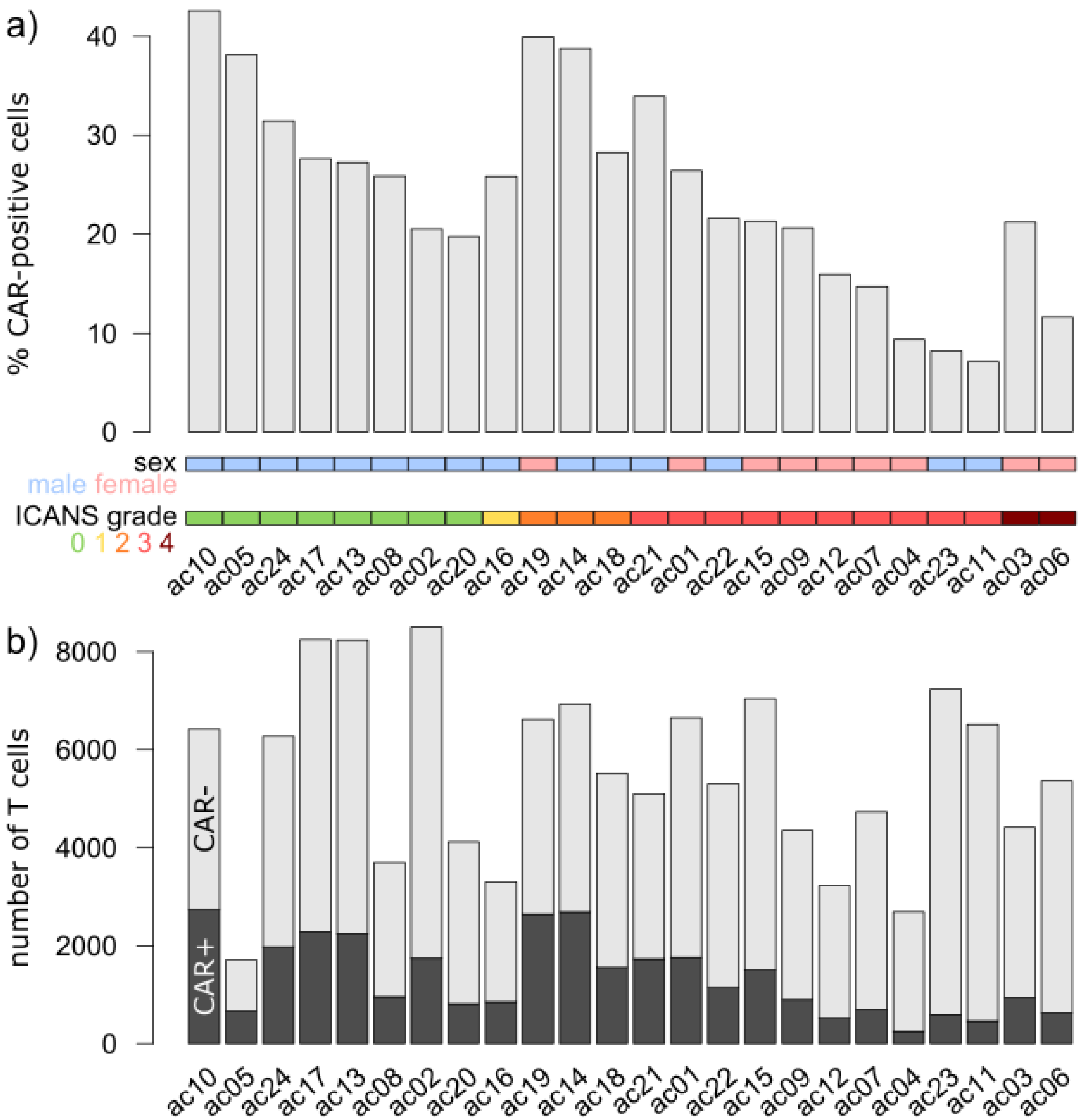

Figure S 4: Proportion of CAR-positive cells and total number of T cells in each of the participants with increasing ICANS grade. a) The percentage of CART+ cells decays, on the average, with ICANS grade. We stratified them into grade 0-2 and grade 3-4 for further analysis (see main paper). Proportion of men is lower in the latter group. b) We found no association of total T cell number and ICANS grade ($p=0.51$ in linear model).

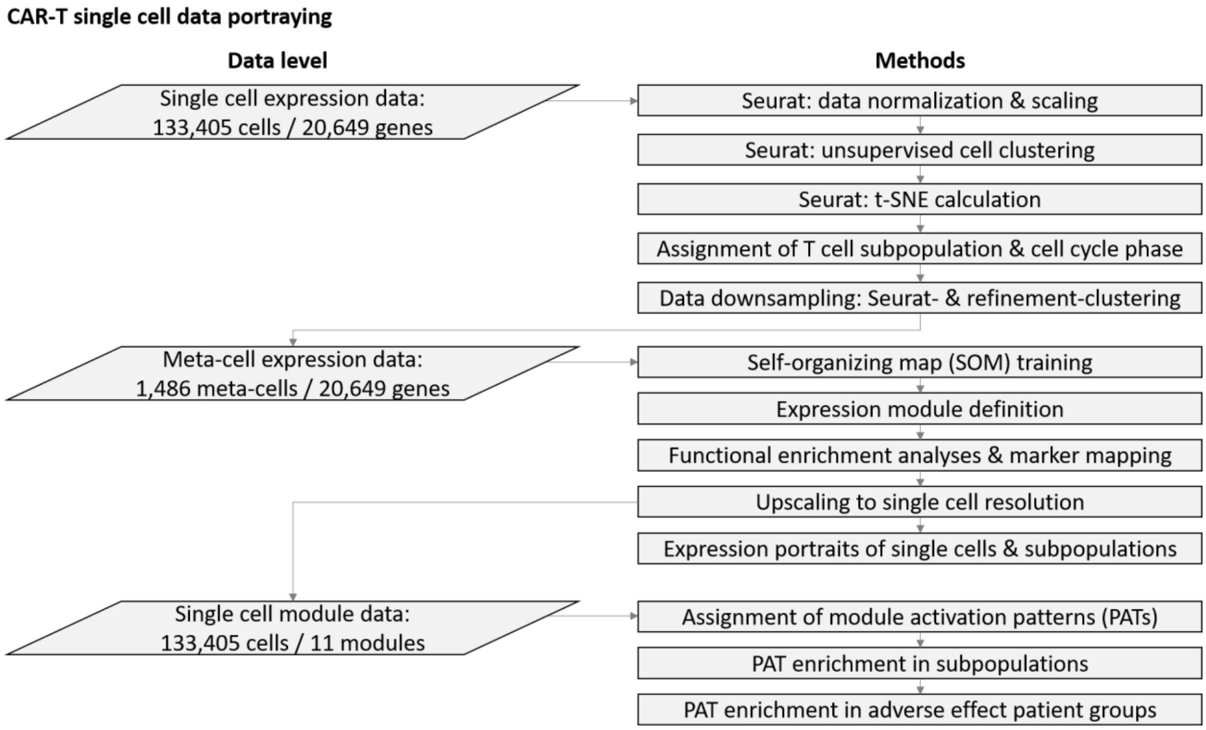

Figure S 5: Flow chart of the CAR-T single cell data portraying workflow: The left column depicts the three different data levels, the right one the methods and algorithms applied. Details are given in the methods section of the main article and the supplementary methods.

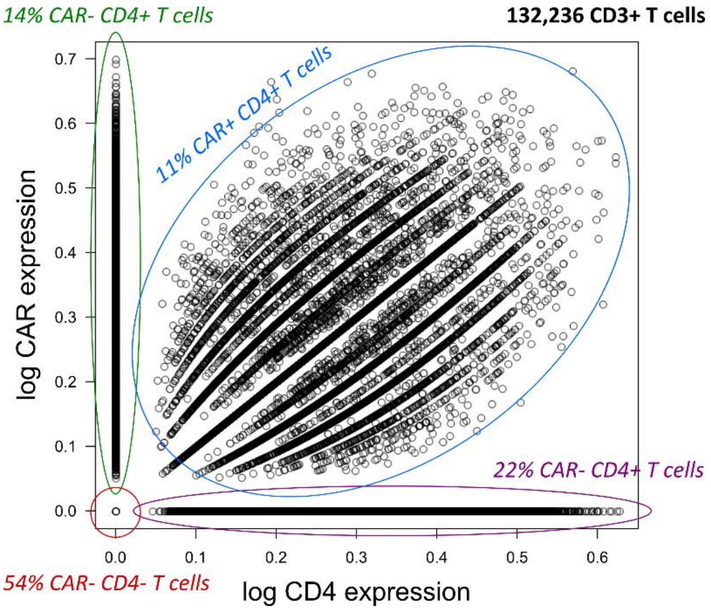

Figure S 6: CD4 and CAR expression characteristics in CD3+ T cells: Expression of CD4 gene and CAR (FMC63-CD19scFV marker) in each cell are used as x- and y-coordinates, respectively. Proportions given in the figure relate to the total number of 132,236 CD3+ T cells in the data set. It shows that CAR gene transfer is measurable in about 34% of CD4+ T cells.

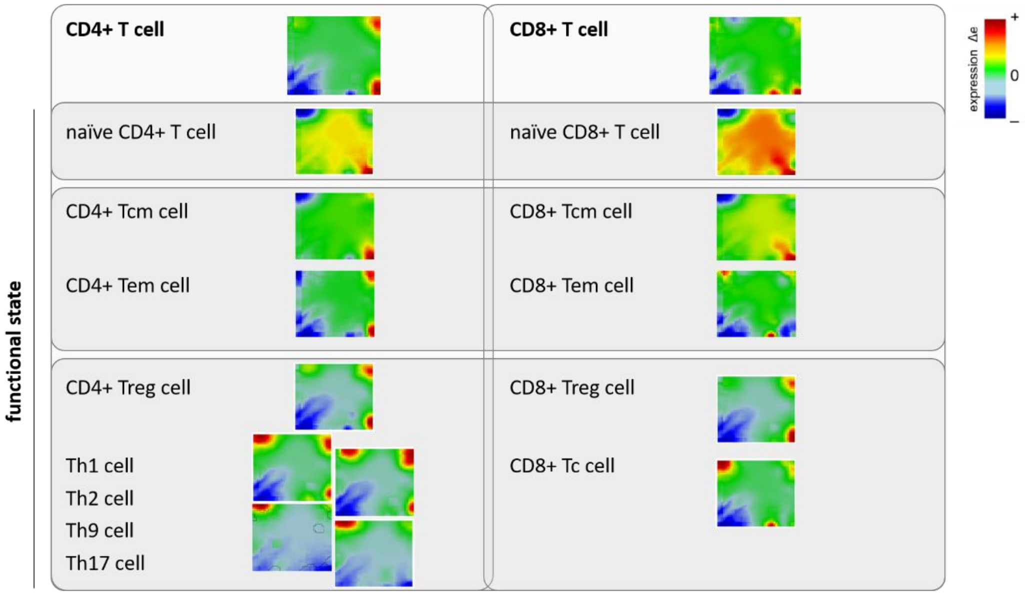

Figure S 7: Gallery of expression portraits of the T cell subpopulations: Meta-genes over-expressed in a subpopulation are shown in red, indifferent ones in green and under-expressed meta-genes in blue colors. Note that the expression portraits can be directly compared as the mapping of the genes to the meta-genes is fixed in all portraits.

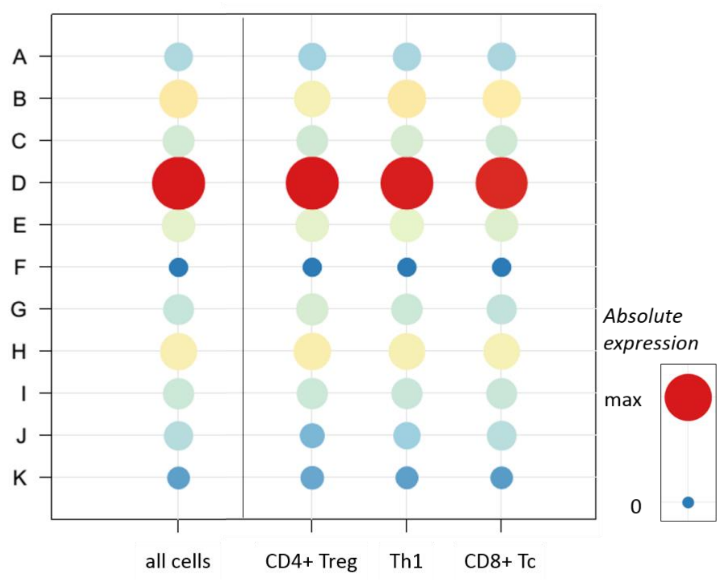

Figure S 8: Mean module overall expression level averaged over all T cells, and grouped by subpopulation (right part), respectively. Blue and red dots represent lowest and highest expression levels, respectively.

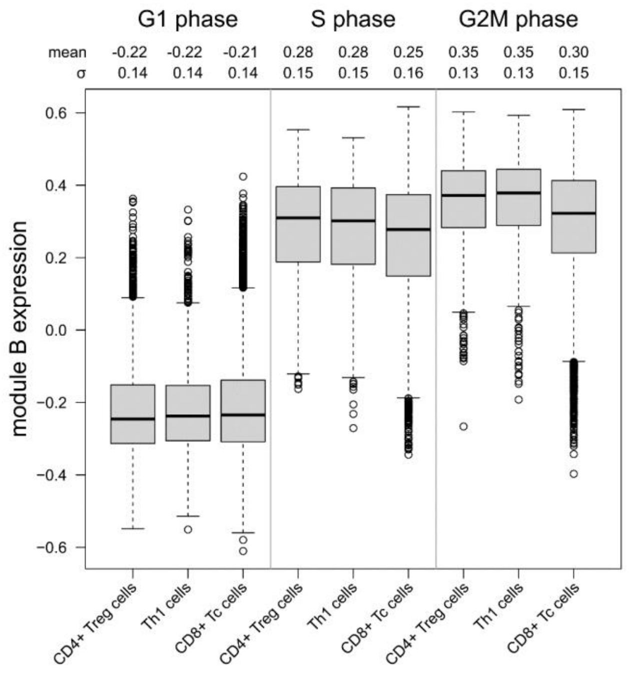

Figure S 9: Expression of module B in single cells: Cells are grouped according to the three major subpopulations and to cell cycle phase. Mean expression value and corresponding standard deviation are given for each of the groups in the header of the figure. Standard deviations exceed differences between the subpopulations by about one order of magnitude.

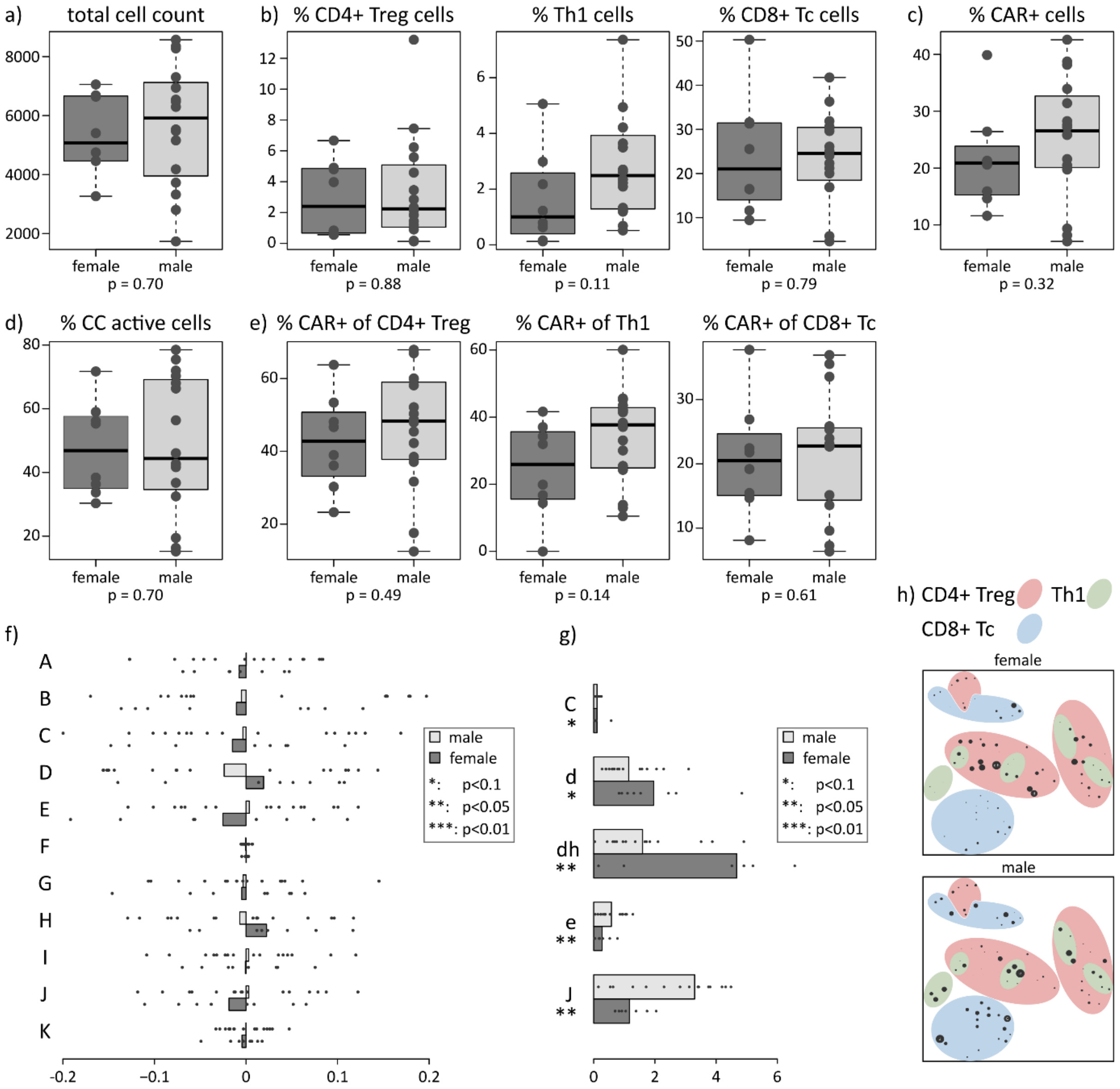

Figure S 10: Comparison of infusion product composition and single cell expression patterns between female and male patients: a) Total number of cells per patient contained in the data set, grouped by sex. P-values are derived from Wilcoxon rank-sum test throughout this figure. b) Relative amount of CD4+ Treg, Th1, and CD8+ Tc cells. c) Relative amount of CAR+ cells. d) Relative amount of cycling genes. e) Relative amount of CAR+ cells in the CD4+ Treg, Th1, and CD8+ Tc subpopulations. f) Mean module expression averaged over all cells derived from male and female patients, respectively. Note that no module was found significantly differential (all p-values > 0.1). g) Relative frequency of the PATs (only PATs with p-value <0.1 are shown). Bar lengths represent the mean percentage for the sexes, the dots represent the individual patients. h) Maps of enriched PATs in cells grouped by sex. T cell subpopulations are highlighted in agreement to the figures in the main article.

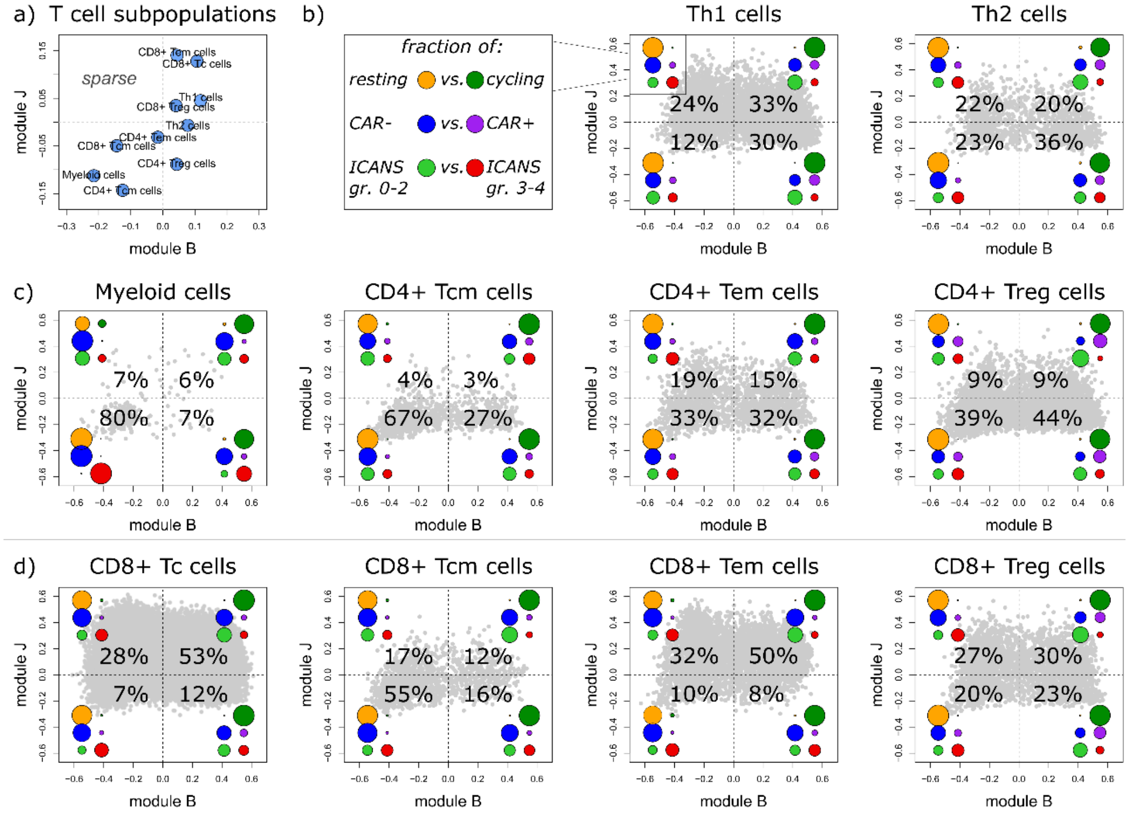

Figure S 11: LAG3-exhaustion score (module J) as a function of the cell-cycle score (module B): a) Mean expression averaged over each T cell type shows positive correlation between the exhaustion and the cell cycle score with highest values for CD8+Tem and Tc cells and lowest values for myeloid and CD4+Tcm cells. b) – d) Distribution of single cells of different T cell types was counted in each of the quadrants (thin crosshair; see fractions of cells in the quadrants), and further stratified according to cell cycle, CAR, and ICANS status (see legend in b). The size of the circles scales with the number of cells in the respective quadrant. In general, T-cell states distribute over the four quadrants, indicating that all four combinations of module J/module B expression (low/low, low/high, high/low, high/high) are high in number. Along the B-axis (left versus right), resting (G1) cells (orange-colored circles) are replaced by cycling (S/G2M) cells (green circles), paralleled by an increased amount of CAR+ cells (purple) and ICANS 0-2 associated cells (light green) in CD4+ and CD8+Treg subpopulations as described in the main manuscript. Along the exhaustion axis (from below to top), one finds an increasing fraction of ICANS 0-2 CD8+Tcm and Tem cycling cells. The majority of myeloid cells (panel c) associate high ICANS with resting and low LAG3-exhaustion.

3 Supplementary tables

Table S 1: Patient characteristics.

Patient ID	Histolog. diagnosis	Sex	ECOG status	Stage	IPI score	therapy lines	refractory	ICANS grade	CRS grade
ac01	DLBCL	female	1	III/IV	2	2	yes	3	2
ac02	DLBCL	male	1	III/IV	1	11	yes	0	2
ac03	DLBCL	female	1	III/IV	2	3	yes	4	4
ac04	DLBCL	male	1	III/IV	4	9	yes	3	3
ac05	tFL	male	1	III/IV	1	5	yes	0	1
ac06	PMBCL	female	1	III/IV	3	4	yes	4	2
ac07	DLBCL	female	1	III/IV	2	4	no	3	1
ac08	PMBCL	male	0	I/II	1	6	yes	0	2
ac09	DLBCL	female	0	III/IV	2	4	yes	3	2
ac10	tFL	male	1	III/IV	3	15	yes	0	2
ac11	tFL	male	1	III/IV	3	7	yes	3	2
ac12	DLBCL	female	1	I/II	1	5	yes	3	3
ac13	DLBCL	male	1	I/II	1	4	yes	0	1
ac14	DLBCL	male	1	III/IV	4	2	yes	2	2
ac15	DLBCL	female	1	I/II	2	4	yes	3	2
ac16	DLBCL	male	1	III/IV	2	4	yes	1	2
ac17	tFL	male	0	III/IV	4	6	yes	0	1
ac18	DLBCL	male	1	III/IV	3	5	yes	2	1

ac19	tFL	female	0	III/IV	4	6	yes	2	1
ac20	DLBCL	male	0	III/IV	3	6	yes	0	1
ac21	DLBCL	male	3	III/IV	3	3	yes	3	3
ac22	DLBCL	male	4	III/IV	3	5	yes	3	1
ac23	tFL	male	0	III/IV	3	4	yes	3	2
ac24	DLBCL	male	0	III/IV	1	2	yes	0	2

Table S 2: Number of genes associated to the expression modules and number of cells with module expression exceeding one standard deviation.

**Module**	**# genes**	**# cells**	
A	89	20,499	(≙ 15.4 % of all cells)
B	339	50,666	(38.0 %)
C	401	26,951	(20.2 %)
D	172	24,173	(18.1 %)
E	152	13,754	(10.3 %)
F	159	96	(0.1 %)
G	266	10,478	(7.9 %)
H	325	20,812	(15.6 %)
I	192	9,437	(7.1 %)
J	123	23,406	(17.5 %)
K	182	1,457	(1.1 %)

Table S 3: T cell subpopulations assigned using the consensus marker scheme. Poly-functional cells are counted multiply.

**T cell subpopulation**	**# cells**	
CD4+ cells	43,111	(≙ 32%)
- *naïve CD4+ cells*	*131*	*(< 1%)*
- *CD4+ Tcm cells*	*1,275*	*(< 1%)*
- *CD4+ Tem cells*	*2,023*	*(2%)*
- *CD4+ Treg cells*	*7,036*	*(5%)*
- *Th1 cells*	*6,386*	*(5%)*
- *Th2 cells*	*1,373*	*(1%)*
- *Th9 cells*	*19*	*(< 1%)*
- *Th17 cells*	*1,046*	*(< 1%)*
CD8+ cells	69,976	(52%)
- *naïve CD8+ cells*	*393*	*(< 1%)*
- *CD8+ Tcm cells*	*1,571*	*(1%)*
- *CD8+ Tem cells*	*4,917*	*(4%)*
- *CD8+ Treg cells*	*3,333*	*(2%)*
- *CD8+ Tc cells*	*39,414*	*(30%)*
CD4+ CD8+ cells	8,003	(6%)

Table S 4: Regression analysis of ICANS grade depending on the proportion of CAR+ cells in the subpopulations adjusted for gender. Models are trained for all patients adjusted for gender, and separately for male and female patients, respectively. In all models, higher CAR+ proportion is associated with lower ICANS grade.

**T cell subpopulation**	**all patients [a, b]**	**male only [b]**	**female only [b]**
CD4+ cells	-6.11 ***	**	*
- *naïve CD4+ cells*	-0.27		
- *CD4+ Tcm cells*	-5.51 ***	**	
- *CD4+ Tem cells*	-5.12 **	**	
- *CD4+ Treg cells*	-5.72 ***	***	*
- *Th1 cells*	-3.54 **	*	
- *Th2 cells*	-4.58 ***	***	***
- *Th9 cells*	-1.01		
- *Th17 cells*	-1.13		
CD8+ cells	-6.61 ***	*	***
- *naïve CD8+ cells*	-0.25		
- *CD8+ Tcm cells*	-2.42		
- *CD8+ Tem cells*	-3.74		***
- *CD8+ Treg cells*	-3.81 **	*	*
- *CD8+ Tc cells*	-6.23 **	*	**

[a] Coefficient (slope) of *ICANS grade* variable in linear regression model

[b] Significance level derived from linear regression model. *: p<0.1; **: p<0.05; ***: p<0.01

4 References

1. Stuart T, Butler A, Hoffman P, Hafemeister C, Papalexi E, Mauck WM, et al. Comprehensive Integration of Single-Cell Data. Cell [Internet]. 2019 Jun 13 [cited 2021 May 3];177(7):1888-1902.e21. Available from: https://pubmed.ncbi.nlm.nih.gov/31178118/

2. Deng Q, Han G, Puebla-Osorio N, Ma MCJ, Strati P, Chasen B, et al. Characteristics of anti-CD19 CAR T cell infusion products associated with efficacy and toxicity in patients with large B cell lymphomas. Nat Med [Internet]. 2020 Dec 1 [cited 2021 Apr 1];26(12):1878–87. Available from: https://pubmed.ncbi.nlm.nih.gov/33020644/

3. Hinton G, Roweis S. Stochastic neighbor embedding. In: Advances in Neural Information Processing Systems. 2003.

4. Sade-Feldman M, Yizhak K, Bjorgaard SL, Ray JP, de Boer CG, Jenkins RW, et al. Defining T Cell States Associated with Response to Checkpoint Immunotherapy in Melanoma. Cell [Internet]. 2018 Nov 1 [cited 2021 Apr 1];175(4):998-1013.e20. Available from: https://pubmed.ncbi.nlm.nih.gov/30388456/

5. Calderon D, Nguyen MLT, Mezger A, Kathiria A, Müller F, Nguyen V, et al. Landscape of stimulation-responsive chromatin across diverse human immune cells. Nat Genet [Internet]. 2019 Oct 1 [cited 2021 Apr 1];51(10):1494–505. Available from: https://pubmed.ncbi.nlm.nih.gov/31570894/

6. Aran D, Hu Z, Butte AJ. xCell: Digitally portraying the tissue cellular heterogeneity landscape. Genome Biol. 2017;18(1).

7. Charoentong P, Finotello F, Angelova M, Mayer C, Efremova M, Rieder D, et al. Pan-cancer Immunogenomic Analyses Reveal Genotype-Immunophenotype Relationships and Predictors of Response to Checkpoint Blockade. Cell Rep. 2017;18(1):248–62.

8. Golubovskaya V, Wu L. Different Subsets of T Cells, Memory, Effector Functions, and CAR-T Immunotherapy. Cancers (Basel) [Internet]. 2016 Mar 15;8(3):36. Available from: http://www.mdpi.com/2072-6694/8/3/36

9. Mahnke YD, Brodie TM, Sallusto F, Roederer M, Lugli E. The who's who of T-cell differentiation: Human memory T-cell subsets. European Journal of Immunology. 2013.

10. Kohonen T. The self-organizing map. Proc IEEE [Internet]. 1990 [cited 2010 Sep 13]; Available from: http://ieeexplore.ieee.org/xpls/abs_all.jsp?arnumber=58325

11. Loeffler-Wirth H, Kreuz M, Hopp L, Arakelyan A, Haake A, Cogliatti SB, et al. A modular transcriptome map of mature B cell lymphomas. Genome Med [Internet]. 2019 Dec 30 [cited 2019 May 22];11(1):27. Available from: https://genomemedicine.biomedcentral.com/articles/10.1186/s13073-019-0637-7

12. Schmidt M, Hopp L, Arakelyan A, Kirsten H, Engel C, Wirkner K, et al. The Human Blood Transcriptome in a Large Population Cohort and Its Relation to Aging and Health. Front Big Data [Internet]. 2020 Oct 30 [cited 2021 Apr 1];3. Available from: https://pubmed.ncbi.nlm.nih.gov/33693414/

13. Kunz M, Löffler-Wirth H, Dannemann M, Willscher E, Doose G, Kelso J, et al. RNA-seq analysis identifies different transcriptomic types and developmental trajectories of primary melanomas. Oncogene [Internet]. 2018 Nov 11 [cited 2018 Aug 3];37(47):6136–51. Available from: http://www.nature.com/articles/s41388-018-0385-y

14. Binder H, Willscher E, Loeffler-Wirth H, Hopp L, Jones DTW, Pfister SM, et al. DNA methylation, transcriptome and genetic copy number signatures of diffuse cerebral WHO grade II/III gliomas resolve cancer heterogeneity and development. Acta Neuropathol Commun [Internet]. 2019 Apr 25 [cited 2019 May 22];7(1):59. Available from: https://actaneurocomms.biomedcentral.com/articles/10.1186/s40478-019-0704-8

15. Camp JG, Sekine K, Gerber T, Loeffler-Wirth H, Binder H, Gac M, et al. Multilineage communication regulates human liver bud development from pluripotency. Nature [Internet]. 2017 Jun 14 [cited 2018 Jan 12];546(7659):533–8. Available from: http://www.ncbi.nlm.nih.gov/pubmed/28614297

16. Löffler-Wirth H, Kalcher M, Binder H. oposSOM: R-package for high-dimensional portraying of genome-wide expression landscapes on Bioconductor. Bioinformatics [Internet]. 2015 Jun 10 [cited 2015 Jun 14]; Available from: http://www.ncbi.nlm.nih.gov/pubmed/26063839

17. Nikoghosyan M, Loeffler-Wirth H, Davidavyan S, Binder H, Arakelyan A. Projection of High-Dimensional Genome-Wide Expression on SOM Transcriptome Landscapes. BioMedInformatics [Internet]. 2021 Dec 27;2(1):62–76. Available from: https://www.mdpi.com/2673-7426/2/1/4

18. Boser BE, Guyon IM, Vapnik VN. A training algorithm for optimal margin classifiers. In: Proceedings of the fifth annual workshop on Computational learning theory - COLT ’92 [Internet]. New York, New York, USA: ACM Press; 1992. p. 144–52. Available from: http://portal.acm.org/citation.cfm?doid=130385.130401
